# Molecular Simulations of Polymer‐based Drug Nanocarriers: From Physical and Structural Properties to Controlled Release

**DOI:** 10.1002/adhm.202503503

**Published:** 2025-09-26

**Authors:** Ping Gao, Xin Jiang, Jinyu Li, Julien Nicolas, Tâp Ha‐Duong

**Affiliations:** ^1^ Fuzhou University College of Chemistry Fuzhou 350116 China; ^2^ Université Paris‐Saclay CNRS, BioCIS Orsay 91400 France; ^3^ Université Paris‐Saclay CNRS Institut Galien Paris‐Saclay Orsay 91400 France

**Keywords:** controlled drug release, drug delivery system, nanoparticle molecular simulation, polymer‐based nanocarrier, polymer molecular modeling

## Abstract

Polymer‐based drug delivery systems have been extensively studied to overcome the limitations of free drug administration (e.g., poor solubility and stability, rapid degradation and early metabolization, short plasma half‐life, low therapeutic efficacy, and occurrence of side effects). Although the vast majority of these drug delivery systems are developed using the traditional time‐ and resource‐intensive trial‐and‐error method, computational techniques have received considerable attention in order to facilitate and accelerate their understanding and development. In this review, several computational techniques is presented that are commonly used to study polymer‐based drug delivery systems. Then, this is discussed several computational investigations of the self‐assembly and supramolecular organization of polymer nanocarriers for drug delivery applications, including drug‐loaded polymer micelles and polymer prodrug nanoparticles. How modeling approaches can rationalize the drug loading and release from polymer drug delivery systems is further examined, including studies which aim to better understand how physical, chemical, or biological stimuli can trigger the drug release. Machine learning possibilities for extending physics‐based molecular simulation efficiency and predictive power have also been briefly discussed.

## Graphical abstract

Polymer‐based drug delivery systems can effectively overcome the limitations of free drugs in terms of solubility, stability, and plasma half‐life, yet their development has traditionally relied on time‐consuming trial‐and‐error approaches. This review highlights recent advances in applying molecular simulation to the design of polymer‐based drug nanocarriers, with a particular emphasis on their self‐assembly, supramolecular organization, drug loading, and interaction mechanisms, as well as the role of stimuli‐responsiveness in modulating polymer nanocarrier structures and drug release. The emerging opportunities for integrating machine learning to enhance simulation efficiency and predictive power have also been discussed.

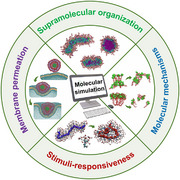

## 1 Introduction

In pharmaceutical developments, many drugs exhibit poor bioavailability, unfavorable pharmacokinetics, or do not hit their targets, resulting in low efficacy and/or severe side effects. To tackle these issues, polymers have attracted great interest as building blocks for the design of polymer nanocarriers as advanced drug delivery systems.^[^ ^1^, ^2^, ^3^, ^4^, ^5^, ^6^, ^7^ ^]^ Indeed, nanocarriers made of (bio)degradable, biocompatible, and non‐immunogenic polymers can be loaded with drugs for improving their solubility and stability against proteases, leading to enhanced bioavailability. Polymer‐based nanocarriers can also increase in vivo circulation time and enhance drug transport across biological barriers and controlled release, achieving better targeting of diseased tissues, thus reducing adverse effects and improving therapeutic efficacy.^[^ ^5^, ^8^, ^9^, ^10^, ^11^, ^12^ ^]^ Many polymers are used for this purpose, such as poly(ethylene glycol) (PEG),^[^ ^13^, ^14^, ^15^, ^16^, ^17^ ^]^ aliphatic polyesters such as polylactide (PLA)^[^ ^18^, ^19^, ^20^ ^]^ and poly(lactide‐*co*‐glycolide) (PLGA),^[^ ^21^, ^22^, ^23^, ^24^ ^]^ polysaccharides^[^ ^25^, ^26^, ^27^ ^]^ and synthetic polypeptides.^[^ ^28^, ^29^ ^]^ Vinyl polymers can also be made (bio)degradable by radical ring‐opening polymerization (rROP) of conventional vinyl monomers with cyclic monomers that are precursors of labile groups (e.g., ester, thioester, disulfide) into the polymer backbone once they are incorporated.^[^ ^30^, ^31^, ^32^, ^33^, ^34^ ^]^

Among the polymer‐based nanocarriers, those that encapsulate drugs non‐covalently are of great interest and widely used in pharmaceutical developments. Nanocarriers with micellar organization and/or a core‐shell morphology can load drugs (especially poorly water‐soluble ones)^[^ ^35^ ^]^ into their core and can be surface‐modified to improve their pharmacokinetics, biodistribution, and targeting efficiency.^[^ ^36^, ^37^, ^38^ ^]^ Aliphatic polyesters have long been considered as one of the ideal choices in this field due to their biocompatibility and biodegradability.^[^ ^39^, ^40^ ^]^ A representative example of such nanocarriers, first reported by Langer in 1994,^[^ ^41^ ^]^ are biodegradable and stealth nanoparticles made of poly(ethylene glycol)‐*block*‐polyester diblock copolymers such as poly(ethylene glycol)‐*block‐*poly(lactic‐*co*‐glycolic acid) (PEG‐*b*‐PLGA) and poly(ethylene glycol)‐*block*‐poly(*ϵ*‐caprolactone) (mPEG‐*b*‐PLC).^[^ ^42^, ^43^ ^]^ Remarkably, docetaxel‐loaded PEG‐*b*‐PLGA nanoparticles surface‐functionalized with targeting ligands have shown success in the first phases of clinical trials.^[^ ^44^ ^]^

Another type of polymer‐based nanocarrier relies on the chemical coupling of the drug to the polymer scaffold. This strategy was first proposed by Ringsdorf in 1975,^[^ ^45^ ^]^ and played an important role in the development of current polymer prodrugs or polymer‐drug conjugates.^[^ ^6^, ^46^, ^47^, ^48^, ^49^ ^]^ Depending on the nature of the polymer and the drug, polymer prodrugs can either be fully water‐soluble or self‐assembled into nanoparticles. In both cases, polymer prodrugs improve the aqueous solubility of hydrophobic drugs, prolong the drug circulation time, increase their bioavailability and can achieve specific targeting.^[^ ^46^, ^50^ ^]^ Typical examples of polymer prodrugs are for instance those based on poly(*N*‐(2‐hydroxypropyl)methacrylamide) (HPMA), designed by Kopeček^[^ ^51^ ^]^ and poly(amino acid)‐based block copolymer nanoparticles, developed by Kataoka,^[^ ^52^ ^]^ which reached phase II and phase II/III clinical trials, respectively.

However, even though significant proofs of concepts using polymer‐based drug nanocarriers have been reported, many of them suffer from significant limitations, which may explain many failures in clinical trials and the still low number of marketed nanoscale drug delivery systems.^[^ ^53^ ^]^ For instance, non‐covalent drug‐loaded polymer nanoparticles generally suffer from the “burst release”, during which a large fraction of encapsulated drugs is quickly released post‐administration, which may lead to prohibitive in vivo toxicity and reduced therapeutic efficacy. The low miscibility between certain drugs and polymers can also alter the colloidal stability of the nanoparticles, thus requiring the use of additional organic solvents. Finally, the drug loadings obtained are often low, requiring the administration of high concentrations of nanoparticles to reach therapeutic doses. Regarding polymer prodrugs, the covalent linkage between the drug and the polymer (also called the linker) plays a key role in the fate of the prodrugs and therefore their therapeutic efficacy. This requires upstream development of chemical/macromolecular synthesis strategies, as the linker's lability and susceptibility to cleavage need to be carefully tuned to ensure effective cleavage at the right time and in the right place (e.g., tumor microenvironment).^[^ ^6^, ^49^, ^54^ ^]^

The physicochemical and nanostructural properties of polymer‐based drug nanocarriers, including size,^[^ ^55^, ^56^ ^]^ shape,^[^ ^57^, ^58^ ^]^ stiffness,^[^ ^59^ ^]^ surface functionalization^[^ ^60^, ^61^, ^62^ ^]^ or surface charge,^[^ ^56^, ^63^ ^]^ are known to have a major impact on the nanocarrier diffusivity in blood vessels, membrane permeability, as well as on the in vivo drug release kinetics and their ability to reach their target.^[^ ^64^, ^65^, ^66^ ^]^ For instance, it has been shown that the tumor penetration and anticancer efficacy of PEGylated polymer micelles loaded with platinum‐based drugs were strongly influenced by their size, as only the 30 nm micelles penetrated poorly permeable pancreatic tumors to achieve significant anticancer efficacy conversely to the 50–100 nm counterparts.^[^ ^67^ ^]^ Functionalizing the surface with a targeting ligand can also have a considerable impact on the fate of polymer‐based nanoparticles to improve their accumulation in disease‐affected areas and optimize therapeutic efficacy.^[^ ^5^ ^]^

When developing new drug delivery systems, all these physicochemical and nanostructural parameters must be precisely determined and carefully adjusted for more efficient development and batch‐to‐batch reproducibility, in order to accelerate preclinical development. They are usually assessed by routine characterization techniques, including dynamic light scattering, (cryogenic) transmission electron microscopy, X‐ray photoelectron spectroscopy (XPS) or small‐angle X‐ray scattering (SAXS).^[^ ^68^, ^69^ ^]^ However, these characterization techniques have intrinsic experimental limitations (e.g., sensitivity, precision, specific operating conditions) and, for some of them, tedious operating conditions and sample processing. In addition, they may not give access to certain essential information, such as the supramolecular organization of nanoparticles, and in particular the internal distribution of drug molecules and other key components (e.g., linkers for polymer prodrugs).

Molecular modeling and simulation techniques play an important role in nanotechnology by predicting the behavior of biomacromolecules at a nanometric scale.^[^ ^70^, ^71^, ^72^, ^73^, ^74^, ^75^, ^76^ ^]^ In nanomedicine and drug delivery, molecular modeling and simulation techniques are of great interest as complementary tools to experimental measurements,^[^ ^77^, ^78^, ^79^, ^80^, ^81^ ^]^ to better characterize drug delivery systems and better understand their dynamic behavior and supramolecular organization, by compensating for the lack of detail due to the limited spatial resolution of experimental characterization techniques. They are also able to predict the impact of modulating the nature of their constituents on the nanocarrier properties and biological performances.^[^ ^79^, ^82^, ^83^, ^84^, ^85^, ^86^ ^]^ Since, the traditional trial‐and‐error strategy used for the development of new polymer‐based drug nanocarriers, is often laborious, costly and time‐consuming, the introduction of computational technologies can therefore rationalize and accelerate the development of more efficient polymer‐based drug nanocarriers.

The aim of this review is to offer insights into the development of computer‐aided research approaches for the rational design of polymer‐based drug delivery systems with optimized pharmacological and therapeutic properties. Herein, we covered and discussed recent advances in the development of drug‐loaded polymer‐based nanocarriers assisted by computational techniques, obtained either by physical encapsulation of drugs or by chemical conjugation of drugs to polymers. We first introduced the different models used (from all‐atom (AA) to coarse‐grained (CG) models) and the main simulation techniques employed for studying the structures and dynamics of polymer‐based drug delivery systems, including Monte Carlo (MC) and molecular dynamics (MD) simulations. We then discussed recent computational studies aimed at investigating the physicochemical, structural and dynamic properties of polymer‐based drug delivery systems, in particular how the nature of the polymers and drugs affects the self‐assembly process, drug distribution and surface properties, which are factors that govern the biological fate. The effect of representative endogenous/exogenous stimuli on drug release is also covered. Finally, we provide a perspective on the challenges and opportunities that machine learning (ML) present for conventional molecular modeling techniques in the study of polymer‐based drug nanocarriers.

## 2 Modeling and Simulation Techniques for Polymer‐Based Drug Nanocarriers

Molecular simulations are powerful tools that can be considered as ultra‐resolution virtual microscopes for studying structural and dynamics properties of soft matter at the atomic level. A wide range of molecular models, ranging from AA to CG to mesoscale models, have been developed to simulate polymer‐based drug nanocarriers on appropriate length and relevant time scales, focusing mainly on polymer‐drug interactions, polymer self‐assembly, drug loading/release from polymer‐based nanocarriers.^[^ ^87^, ^88^ ^]^ For each model, several simulation techniques can be used, including MC or MD. Although molecular docking has been sometimes used to estimate polymer‐drug interactions and binding energies, its applicability to large and dynamics molecular systems, such as polymer‐based drug nanocarriers, is severely limited and will not be discussed herein. In this section, we describe several molecular models commonly used in polymer‐based drug nanocarriers, the principles of the simulation techniques used in association with these models, as well as the information and new perspectives to which they give access.

### 2.1 Molecular Models of Polymer‐Based Drug Nanocarriers

**Figure 1:**
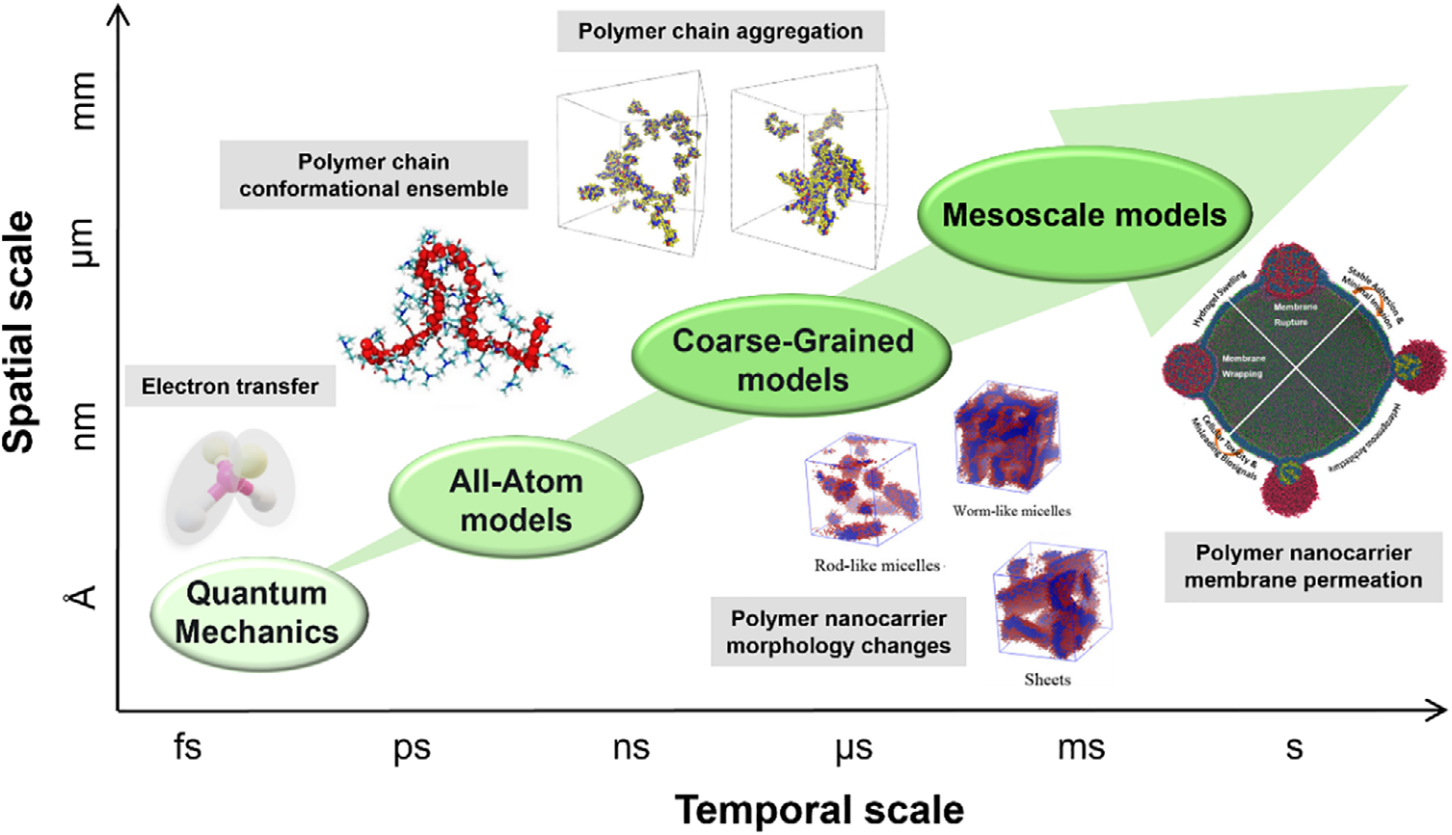
Schematic diagram showing the spatial and temporal scales of molecular modeling approaches for different polymer‐based systems. Green ellipses indicate characteristic regimes where a specific modeling approach is most appropriate. Quantum mechanics (QM) describes electronic processes such as electron transfer. AA models capture polymer chain conformational dynamics. Reproduced with permission.^[^ ^89^ ^]^ Copyright 2017, Elsevier. CG models enable efficient modeling of polymer chain self‐assembly. Reproduced with permission.^[^ ^90^ ^]^ Copyright 2021, American Chemical Society. Mesoscale models can describe phenomena such as morphological changes of polymer‐based nanocarriers, reproduced with permission.^[^ ^91^ ^]^ Copyright 2016, American Chemical Society, and their membrane permeation, reproduced with permission.^[^ ^92^ ^]^ Copyright 2021,American Chemical Society.

The models used for macromolecules can be classified according to the size of the studied systems (from nanometers to micrometers) and the time scale of the investigated processes (from nanoseconds to microseconds). AA models accurately describe the atomic interactions governing the molecular behaviors but due to their high computational cost, they are rarely used to study the self‐assembly of several polymer chains. CG models cannot accurately account for the local structuration of polymer chains, but they are used to study the dynamic process of several chains over long time scales. Mesoscopic models further simplify structural details while retaining the ability to capture morphological transitions of polymer‐based nanocarriers, as well as to provide insights into their interactions with cells (**Figure** **1**). Here, we briefly review the physical basis of these different models and discuss their advantages and limitations, with the aim of informing the rational selection of the most appropriate model for polymer‐based drug delivery systems.

#### 2.1.1 All‐Atom Models

In AA models, all the atoms in the molecules studied, including the hydrogens, are explicitly taken into account and each of them is represented by a particle. The models include a potential energy function (*E_pot_*), also called force field, which describes the physical interactions between all the pairs of atoms, in order to predict the structure and dynamics behavior of the whole molecule. The force fields generally consist of a sum of interaction terms associated with covalent bond stretching (*E_r_*), valence angle bending (*E_θ_*), and torsion angle rotating (*E_ϕ_*), as well as van der Waals (*E_vdw_*) and Coulomb (*E_coul_*) interactions. The most common formulations of these five energy terms are shown in Equations (1), (2), (3), (4), (5), (6).

(1) $$E_{pot}=E_{r}+E_{\theta }+E_{\phi }+E_{vdw}+E_{coul}$$

(2) $$E_{r}=\sum_{bonds}\frac{K_{r}}{2}{\left(r-r_{0}\right)}^{2}$$

(3) $$E_{\theta }=\sum_{angles}\frac{K_{\theta }}{2}{\left(\theta -\theta_{0}\right)}^{2}$$

(4) $$E_{\phi }=\sum_{\mathrm{tors}\mathrm{ions}}\frac{K_{\phi }}{2}\left[1+\cos\left(n\phi \right)\right]$$

(5) $$E_{\mathrm{vdw}}=\sum_{\mathrm{ij}}4\varepsilon_{\mathrm{ij}}\left[{\left(\frac{\sigma_{\mathrm{ij}}}{r_{\mathrm{ij}}}\right)}^{12}-{\left(\frac{\sigma_{\mathrm{ij}}}{r_{\mathrm{ij}}}\right)}^{6}\right]$$

(6) $$E_{\mathrm{coul}}=\sum_{\mathrm{ij}}\frac{q_{i}q_{j}}{4\pi \varepsilon_{0}r_{\mathrm{ij}}}$$

In these equations, all these force field parameters (the quadratic force constants *K_r_* and *K_θ_*, the bond length and valence angle equilibrium values *r*_0_ and *θ*_0_, the barrier heights *K_ϕ_* and multiplicity *n* of the torsion angles, the parameters *ϵ_ij_* and *σ_ij_* of the Lennard‐Jones potentials which account for the van der Waals attraction and short‐range electronic repulsion, as well as the atomic partial charges *q_i_ and q_j_*) are fixed by using experimental data and/or quantum mechanics calculations to reproduce known molecular structures as realistically as possible.

Commonly used AA force fields include the Assisted Model Building with Energy Refinement (AMBER) force field,^[^ ^93^ ^]^ which originally designed for proteins and nucleic acids, is now widely employed for small organic molecules such as drug compounds; the Chemistry at Harvard Macromolecular Mechanics (CHARMM) force field,^[^ ^94^ ^]^ notable for its broad applicability to proteins, lipids, and synthetic polymers; and the Optimized Potentials for Liquid Simulations–All Atom (OPLS‐AA) force field^[^ ^95^ ^]^ known for its accuracy in modeling small molecules and biomolecular systems. These force fields generally share similar functional forms (Equations (1), (2), (3), (4), (5), (6)) but can differ in the implementation of intramolecular interaction corrections—such as the inclusion of Urey‐Bradley terms to model 1,3 interactions in CHARMM—or in the scaling of certain interaction terms in AMBER (e.g., a 0.833 scaling factor for 1,4‐LJ and Coulomb interactions). Unlike traditional AA force field, the Groningen Molecular Simulation (GROMOS) force field^[^ ^96^ ^]^ is a widely used united‐atom (UA) model, which groups nonpolar hydrogens with the carbons to which they are bonded into single interaction sites, significantly reducing the computational cost of simulations. GROMOS has been widely used for biomolecules (especially lipid bilayers),^[^ ^97^ ^]^ however, it can be limited in its ability to reproduce certain experimental properties, and only a few set of lipid types has been thoroughly tested.^[^ ^98^ ^]^ Some GROMOS‐like force fields (e.g., PRODRGFF for small molecules) have been reported to lack accuracy in predicting osmotic coefficients for drug‐like molecules.^[^ ^99^ ^]^ As such, the selection of force fields must be carried out with caution, as inappropriate choices can adversely affect simulation accuracy, transferability, and computational efficiency.

For example, classical parameters from generalized force fields (e.g., OPLS), when applied without system‐specific adjustment, may fail to capture important electronic effects such as conjugation along polymer backbones.^[^ ^100^ ^]^ This issue is further complicated by the markedly different chemical and physical characteristics of drug molecules and polymers, raising critical questions regarding the transferability of force field parameters. To address these limitations, hybrid approaches have been proposed. For instance, PLAFF3^[^ ^101^ ^]^—a force field derived from a combination of OPLS and CHARMM—includes modifications to bonded and nonbonded parameters to better reproduce experimentally observed properties of PLA, such as crystal structure conformations, melt density, volume expansivity, and glass transition temperature. To enable interactions between chemically distinct species, many force fields rely on combination rules, such as the Lorentz‐Berthelot (LB) convention^[^ ^102^ ^]^ to calculate cross‐interaction Lennard‐Jones terms between different atom types. However, it is important to note that force fields are not inherently interchangeable, and mixing parameters derived from different force field families can introduce inconsistencies. These details are beyond the scope of this review, but readers are pointed to several articles on the subject.^[^ ^103^, ^104^ ^]^

In contrast to classical force fields, polarizable force fields (PFFs) account for many‐body polarization effects by allowing atomic charges or dipoles to dynamically respond to the local electrostatic environment.^[^ ^105^ ^]^ This feature enables more accurate modeling of non‐covalent interactions—including hydrogen bonding, ion pairing, and polarization‐induced conformational changes^[^ ^106^ ^]^ which are often critical in biochemical and soft‐matter systems. A representative example is the Atomic Multipole Optimized Energetics for Biomolecular Applications (AMOEBA) force field,^[^ ^107^ ^]^ which emphasizes reproducing molecular polarizabilities and electrostatic potentials rather than solely fitting interaction energies, allowing AMOEBA to more accurately capture structures and thermodynamics of protein‐small molecule complexes. Although polarizable force fields (PFFs) offer improved accuracy in modeling electrostatic interactions, their widespread adoption remains limited due to challenges in parameterization and significantly higher computational cost compared to non‐polarizable counterparts.^[^ ^108^ ^]^ Nevertheless, their integration into multiscale modeling frameworks holds significant promise for future applications in complex biomolecular and polymeric systems.

Overall, although detailed AA simulations can provide valuable atomistic insights into molecular interactions, structures, and dynamics of polymer‐based drug nanocarriers,^[^ ^109^, ^110^, ^111^ ^]^ they generally generate trajectories of limited duration (from hundreds of nanoseconds to a few microseconds). This is partly due to the necessity to explicitly take into account the solvent molecules in the simulations to accurately reproduce the structures, dynamics, and interactions of these molecular systems. Typically, AA simulations employ three‐site rigid water models, such as SPC/E^[^ ^112^ ^]^ or TIP3P,^[^ ^113^ ^]^ to achieve a satisfactory compromise between accuracy and computational efficiency. Nevertheless, flexible or four‐site water models, such as TIP4P‐based models,^[^ ^114^, ^115^ ^]^ may be necessary to more accurately reproduce the electrostatic properties of water, the thermodynamics of solvent, as well as the effects of solvation on the conformational dynamics of polymers, albeit at an even higher computational cost. As a result, slower relevant processes—such as polymer aggregation/dissolution, nanoparticle morphological transitions, drug release, or cellular uptake—may remain inaccessible without the use of enhanced sampling techniques or CG approaches (discussed in subsequent sections).

#### 2.1.2 Coarse‐Grained Models

**Figure 2:**
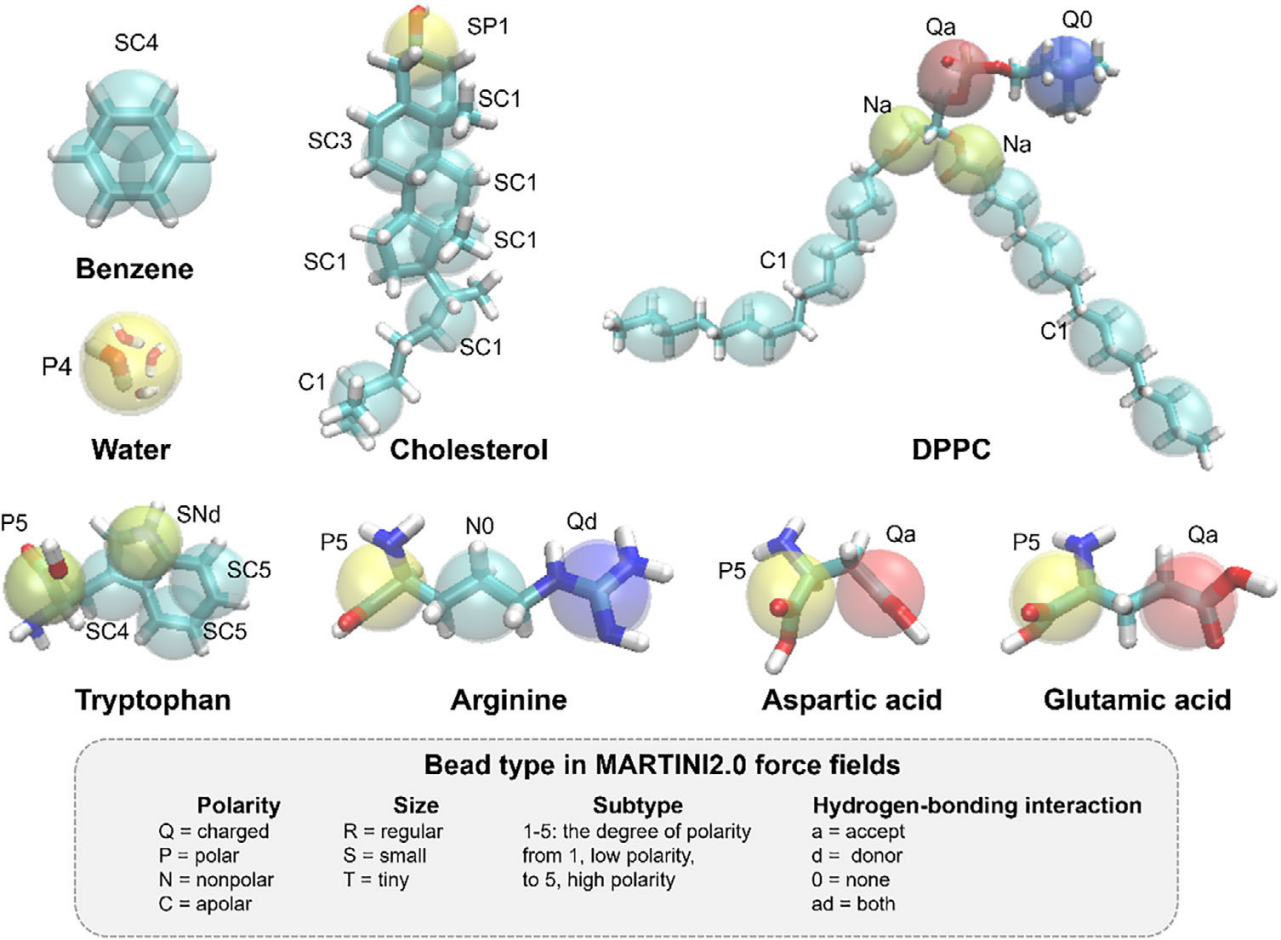
Examples of CG models for various molecules. Each semi‐transparent bead represents a few heavy atoms. Labels indicate the bead chemical property in the MARTINI2.0 force fields.^[^ ^120^ ^]^

CG models allow simulations on time scales 2‐3 orders of magnitude longer than AA models by reducing the number of degrees of freedom of the molecular system.^[^ ^116^ ^]^ This simplification is achieved by grouping atoms into larger interaction sites called “beads”. For example, a polymer chain can be described with one bead representing each monomer unit.^[^ ^117^, ^118^, ^119^ ^]^ Among the various CG models, the MARTINI force field is one of the most widely adopted, primarily due to its top‐down parameterization strategy that reproduces thermodynamic properties such as partition coefficients and solvation free energies, thereby ensuring transferability across diverse molecular systems.^[^ ^120^ ^]^ In MARTINI force fields (**Figure** **2**), the 4‐1, 3‐1, and 2‐1 mapping schemes are employed to map four, three, and two heavy atoms and associated hydrogens, respectively, into a single regular (R), small (S), and tiny (T) bead. Typically, the 4‐1 scheme is the best option for a regular structure to enhance computational performance. In the case of aromatic ring structures, two heavy atoms are mapped into a single bead to represent the flatness of the ring.^[^ ^120^, ^121^ ^]^ These standardized mapping rules and broad chemical coverage make MARTINI highly versatile for biological and soft matter systems. Recent developments within the MARTINI framework, particularly the release of MARTINI 3,^[^ ^122^ ^]^ have further enhanced its performance by introducing a refined bead classification system and a reparametrized interaction matrix, leading to significantly improved accuracy in modeling biomolecular interactions. Importantly, MARTINI 3 force fields also greatly improve previous limitations in secondary structure stability, ion specificity, and the modeling of small drug‐like molecules.^[^ ^123^ ^]^

CG force fields are generally formulated similarly to AA models (Equations (1), (2), (3), (4), (5), (6)), except that the energy terms describe interactions between pairs of beads. Bonded interaction parameters can be determined by reproducing the distributions of the pseudo‐bond distances, pseudo‐valence angles, and pseudo‐dihedral angles extracted from AA simulations of the polymer chains.^[^ ^116^ ^]^ The nonbonded interactions are generally modeled by using Lennard‐Jones‐type functions and Coulomb potentials. The strength of the Lenard‐Jones interactions depends on the hydrophilic/hydrophobic property of the beads. For example, in MARTINI models, there are four main types of beads (charged (Q), polar (P), non‐polar (N), and apolar (C)), and each particle type has a number of subtypes to accurately represent their chemical nature. The depth of their Lennard‐Jones wells varies from 2.0 to 5.6 kJ/mol.^[^ ^120^ ^]^ Electrostatic interactions between charged beads can be modeled using a Coulomb term with Debye‐Hu¨ckel^[^ ^124^ ^]^ screening to account for salt concentration or by Coulomb potentials simply screened with an ad hoc relative dielectric constant (*ϵ_r_* = 15 in MARTINI force fields,^[^ ^120^ ^]^ for instance).

While MARTINI represents a generic coarse‐graining approach based on the use of transferable bead types across a wide range of molecular systems, systematic coarse‐graining methods offer a more tailored and physics‐based framework. The latter employs bottom‐up parameterization strategies, where CG potentials are derived from AA simulations or experimental data to preserve key structural and thermodynamic properties of the system. Representative examples of systematic CG methods include force matching (also known as multiscale coarse‐graining),^[^ ^125^ ^]^ iterative Boltzmann inversion (IBI),^[^ ^126^ ^]^ and Surface Property fItting Coarse grAined (SPICA) model.^[^ ^127^ ^]^ Notably, multiscale modeling frameworks has been demonstrated to be effective in the simulation of membrane proteins^[^ ^128^ ^]^ and polymers,^[^ ^129^ ^]^ however, their transferability may be limited. More recently, machine learning‐based CG models have gained momentum. These models utilize neural networks or graph‐based representations to learn effective interaction potentials that retain atomistic accuracy while providing CG‐level efficiency.^[^ ^130^, ^131^ ^]^ Although this review does not delve into the detailed methodologies or theoretical foundations of these approaches, it is important to note that their system‐specific nature enables the development of chemically accurate CG models, particularly valuable for complex or nonstandard molecular systems, such as metal–organic frameworks.^[^ ^132^ ^]^

Despite the significant extension of accessible spatial and temporal scales and the reduction in computational cost, accuracy remains a central concern in CG modeling. Typically, the CG force field parameters are generally determined to best reproduce quantities that can be measured experimentally, such as the polymer radius of gyration,^[^ ^133^ ^]^ diffusion coefficients, and, in the case of MARTINI force fields, the free energies of vaporization, hydration, and partitioning (e.g., water‐octanol or water‐hexadecane).^[^ ^120^ ^]^ Thanks to a well‐balanced compromise between chemical accuracy and computational efficiency, CG models have become one of the most widely used approaches for simulating large‐scale systems, ranging from multiple polymer chains^[^ ^117^, ^134^ ^]^ and their self‐assembly into nanoparticles/micelles,^[^ ^90^, ^135^, ^136^ ^]^ to more complex interaction mechanisms involved in the delivery process.^[^ ^137^ ^]^ However, the inherent coarse resolution of these models can limit their ability to accurately capture local conformational flexibility, specific hydrogen bonding, and stereochemical effects—features that are especially important when modeling small‐molecule drugs or structurally intricate polymers. Consequently, while CG models offer powerful tools for exploring mesoscale behavior, they must be used with caution when detailed atomistic features are critical for the phenomena under investigation.

#### 2.1.3 Dissipative Particle Dynamics

Dissipative particle dynamics (DPD) is both a molecular model and a simulation technique. This representative mesoscopic modeling method, which describes molecular systems at a lower resolution than CG models, and thus can be uses to study complex hydrodynamic behavior of larger systems (like complex fluids, large polymers aggregates) over longer time scales (*µ*s‐ms) with computational efficiency.^[^ ^138^ ^]^ In DPD modeling, several molecules of fluid or several building blocks of a polymer chain are clustered into one fluid particle. Each particle interacts with the other ones through a pairwise additive force composed of three terms: a conservative soft repulsive force (instead of a hard‐core Lennard‐Jones potential) which intensity depends on the chemical nature of the particles, a dissipative term representing the effects of viscosity, and a random force accounting for the thermal energy of the system.^[^ ^139^ ^]^ Particles can be connected through pseudo‐bonds to form molecules by attaching them to each other with flexible springs like in CG models.^[^ ^140^ ^]^ DPD models are parameterized with bottom‐up procedures by fitting structural features provided by AA simulations^[^ ^141^, ^142^ ^]^ and/or with top‐down approaches by reproducing experimental macroscopic measurements,^[^ ^143^, ^144^ ^]^ such as pressure, density, compressibility, surface tension, etc.

DPD models can effectively investigate molecular packing and dispersion, and study the phase behavior of complex polymer systems (e.g., liquid‐liquid and liquid‐solid phase separation) as well as their mesoscale structural characteristics.^[^ ^140^, ^143^ ^]^ Recent research has demonstrated the great practicability of DPD model in studying the self‐assembly of amphiphilic polymers into micelles.^[^ ^145^, ^146^ ^]^ Furthermore, the DPD models offer significant advantages in studying multi‐component systems comprising polymers and lipid bilayers, which are crucial for understanding membrane translocation or cellular uptake processes of polymer nanoparticles. DPD simulations have also shown the capability to predict microstructures of polymer micelles and their morphological changes,^[^ ^147^, ^148^ ^]^ along with polymer‐drug/payload interactions.^[^ ^149^, ^150^ ^]^ However, microscopic details of polymer‐drug conjugates by DPD models still remain difficult to obtain and many systems are yet to be tested.

### 2.2 Simulation Techniques to Study Polymer‐Based Drug Nanocarriers

This section presents the computational techniques commonly used with the molecular models described above to study polymer‐based drug nanocarriers. These techniques can be divided into three categories: (i) MC simulations, which involve the stochastic sampling of the system configurations; (ii) MD simulations, which are based on solving the Newton's equations of motion of the system particles, and (iii) enhanced MD simulations, which are eventually required for complex biological systems and large materials, to accelerate the conformational phase exploration or to sample rare events, by introducing biases or perturbations to the system. This section presents the principles behind these three types of simulation techniques.

#### 2.2.1 Monte Carlo Simulation

MC simulation is an important technique commonly used to study the thermodynamic properties of molecular liquids, polymers structures, biological systems, and rational drug design.^[^ ^151^, ^152^, ^153^ ^]^ In a typical MC simulation, a random variable of the system (e.g., the position of a particle) is perturbed randomly, and the acceptance of the new configuration is determined probabilistically, based on the change in the system potential energy—often using the Metropolis criterion.^[^ ^154^ ^]^ MC steps are repeated millions of times to produce a statistical ensemble partition function of the system which is used to calculate macroscopic quantities such as free energies. MC simulations are fundamentally designed for sampling equilibrium distributions and calculating state probabilities. Starting from an initial configuration, the system is allowed to evolve until it reaches a steady state, which is characterized by a set of constant thermodynamic properties (e.g., temperature, pressure, energy). MC are widely used methods in materials science and polymers properties, and their extension to the investigation of molecular mechanisms underlying drug delivery has attracted growing interest.^[^ ^155^, ^156^, ^157^, ^158^ ^]^ For instance, the influence of various nanoparticle parameters, including nanoparticle core size, surface functionalization, and grafting density, on the nanoparticle binding energy and their cell‐targeting efficiency has been systematically studied by MC simulations.^[^ ^159^ ^]^ The results indicate that the affinity of nanoparticles for cell membranes can be modified by adaptively modulating the above parameters, thereby achieving targeting specificity.

However, standard MC methods do not provide direct information about the system's time evolution or dynamics. While non‐equilibrium processes can be modeled using specialized variants like kinetic Monte Carlo (kMC)^[^ ^160^ ^]^ which simulate the temporal evolution of systems under external driving forces—these approaches are algorithmically distinct from conventional MC and will not be discussed in detail in this review. It is interesting to note that, in many instances, MC simulations are also employed together in combination with MD simulations or docking calculations for the design of polymer‐based drug delivery systems.^[^ ^161^, ^162^, ^163^ ^]^

#### 2.2.2 Classical Molecular Dynamics Simulation

Classical MD simulation is a computational method which numerically solve the Newton's equations of motion to simulate the dynamic behavior of atoms and molecules over time.^[^ ^164^ ^]^ In brief, the velocity and position of each particle of the system at time *t* + ∆*t* are derived from those at time *t*, its mass, and the force acting on it (through the interaction potential energy function), generating the spatial trajectory of each atom and molecule over time. In AA simulations, the time step ∆*t* is typically a few femtoseconds (10*^−^* ^15^ s) to be smaller than the fastest molecular vibration period.^[^ ^165^ ^]^ Since most of the relevant biomacromolecular events, such as changes in functional structure or associations, generally take place on timescales of the order of microseconds or more, their simulations require several weeks or months, even on high‐performance computers.^[^ ^166^ ^]^ Fortunately, it could be noted that the heavier the particles in the system, the larger the integration time step ∆*t* can be, encouraging the use of CG or DPD models to generate more efficiently trajectories over a long duration.

This way, MD simulations can capture a variety of relevant biomacromolecular processes and provide detailed information on their structure, thermodynamic, and kinetics properties, including conformational changes and ligand binding. In structural biology, MD simulations are often used in complement to various experimental techniques when information are missing or sparse,^[^ ^78^, ^167^ ^]^ notably in the case of intrinsically disordered proteins.^[^ ^168^ ^]^ Molecular simulations can also be used for the interpretation of experimental data.^[^ ^169^, ^170^, ^171^ ^]^ Regarding polymers and drug delivery systems, applications of classical MD simulations have been focused on: (i) the structure and dynamics of polymers, their phase behavior, and mechanical properties;^[^ ^172^, ^173^ ^]^ (ii) the supramolecular organization of polymer‐based drug nanocarriers, their interactions with drugs, lipids, and proteins;^[^ ^162^, ^171^, ^174^ ^]^ and (iii) the mechanisms of drug loading and release to help designing more effective drug delivery systems^[^ ^167^, ^175^ ^]^ (see next section).

#### 2.2.3 Enhanced MD Simulation

To gain insight into biomacromolecular processes that occur on time scales beyond the reach of classical MD simulations, such as protein‐ligand binding, polymer aggregation‐dissociation dynamics, or membrane permeation, enhanced sampling techniques have been developed. These methods help systems to overcome high energy barriers by adding external forces or biases to their potential energy functions, thereby allowing them to explore otherwise inaccessible configurations. Unlike unbiased MD, which samples natural trajectories governed by Newton's equations of motion, enhanced MD accelerates transitions between metastable states, enabling efficient sampling of rare events and slow processes with improved statistical accuracy. A major advantage of enhanced MD is its ability to compute free energy differences that offer deep insight into binding affinities, conformational stability, and reaction mechanisms, all of which are essential in rational drug design and targeted delivery. In the following, we present some of these enhanced MD simulation techniques.

***Steered molecular dynamics*** consists in applying an external force to a molecule or group of atoms to pull it from an initial state toward a different one along a chosen reaction path, and then observing the response of the whole system to mechanical deformation or unbinding events.^[^ ^176^ ^]^ Steered MD has been widely used and is particularly well‐suited for studying mechanical processes or forced transitions, for example, to gain insight into the unfolding of protein domains,^[^ ^177^ ^]^ the binding and unbinding processes of ligands to proteins,^[^ ^178^ ^]^ as well as the translocation of compounds through lipid bilayers, membrane channels, and transporters.^[^ ^179^, ^180^ ^]^ The use of steered MD can greatly accelerate routine simulations in the design of novel drugs and drug delivery systems.^[^ ^181^, ^182^, ^183^ ^]^

***The umbrella sampling*** principle is to run MD simulations of the system which is maintained in some particular configurations along a chosen reaction coordinate by applying bias harmonic constraints.^[^ ^184^ ^]^ By simulating multiple overlapping “windows” along this coordinate and combining the results using the weighted histogram analysis method (WHAM)^[^ ^185^ ^]^ one can reconstruct an unbiased potential of mean force (PMF)—a free energy profile (FEP) that quantifies the stability and transition barriers between states. Umbrella sampling is particularly effective for calculating binding affinities, permeation profiles, and energy barriers associated with molecular entry or exit processes. It provides precise control over sampling and is best suited for systems with a clearly defined reaction coordinate. However, the accuracy and reliability of the results depend critically on the appropriate selection of the reaction coordinate. Umbrella sampling has been used, for instance, to better understand protein‐ligand association mechanisms.^[^ ^186^, ^187^ ^]^ Additionally, it has also been used to investigate the interactions between drugs and polymer nanocarriers^[^ ^188^, ^189^ ^]^ and drug permeation across the cell membrane.^[^ ^190^, ^191^ ^]^

***Metadynamics*** is another powerful enhanced sampling technique to gain insight into the thermodynamics of complex systems as a function of a few collective variables, such as the distance between two groups of atoms, the polymer radius of gyration, or the content of helical/extended residues of a protein. The algorithm accelerates the sampling of rare events by adding a series of Gaussian penalty potentials to the system energy to push it out from local minima.^[^ ^192^ ^]^ Metadynamics is ideal for studying large conformational changes of proteins,^[^ ^193^ ^]^ peptides, and polymers.^[^ ^194^ ^]^ It has also been used to gain insight into the binding/unbinding process and stability of polymer‐drug nanoparticles,^[^ ^195^ ^]^ the drug‐polymer interactions,^[^ ^196^ ^]^ as well as polymer interactions with cell membranes.^[^ ^197^ ^]^

***Replica exchange molecular dynamics*** (REMD) is also frequently used to enhance the sampling of rare events.^[^ ^198^ ^]^ REMD enhances sampling by running multiple MD simulations (replicas) at different temperatures and periodically exchanging configurations between them, allowing systems to overcome energy barriers more efficiently. This REMD variant uses replicas at different temperatures (T‐REMD), while Hamiltonian REMD (H‐REMD) varies interaction potentials to selectively bias specific degrees of freedom. These strategies showed effective conformational sampling than conventional MD in biological systems, such as the membrane penetrability of Alzheimer's peptides.^[^ ^199^ ^]^

***The adaptive biasing force*** (ABF) technique allows to calculate free energy differences through conformational changes.^[^ ^200^ ^]^ ABF dynamically estimates and cancels the mean force along a predefined reaction coordinate, enabling uniform exploration of the free energy landscape, which are especially effective in capturing thermodynamic properties and transition pathways across conformational landscapes. For example, the ABF technique was used to calculate the PMF between paclitaxel (Ptx) and poly(hydroxybutyric‐*co*‐hydroxyvaleric acid) (PHBV) nanoparticles. The high affinity between Ptx and PHB could provide an explanation for the minimal drug release and the relatively low toxicity toward cancer cells.^[^ ^201^ ^]^

Overall, while various modeling frameworks and simulation methodologies offer powerful tools for investigating polymer‐based drug delivery systems, selecting an appropriate approach can be challenging due to differences in resolution, accuracy, and computational cost. In this context, insights from prior studies and established parameterization strategies offer valuable guidance for informed model selection and simulation design. Over the past decades, these computational techniques have significantly advanced our understanding of drug delivery and targeting processes—particularly when combined with experimental data and multiscale hybrid strategies. They have proven instrumental in elucidating the structural organization, drug loading and release mechanisms, and translocation of polymer‐based nanocarriers,^[^ ^77^, ^162^, ^202^, ^203^ ^]^ thereby accelerating the future design of smart and controlled drug delivery systems.

## 3 Computational Studies of Polymer‐Based Drug Delivery Systems

This section reviews the applications of the previously described computational techniques in the investigation of the structural properties of polymer‐based drug delivery systems and the thermodynamic characteristics of their drug delivery process. Particular emphasis is placed on the types of mechanistic insights that these techniques can provide—such as conformational dynamics, binding affinities, free energy profiles, and diffusivity—which are of significant interest to experimental researchers seeking to interpret physical behaviors at the molecular level. Recognizing that detailed theoretical models may appear abstract to non‐specialists, representative case studies are presented to illustrate how these computational strategies can be effectively combined with experimental data to construct simulation‐experiment hybrid frameworks in achieving a deeper understanding of drug loading, release kinetics, and carrier‐drug‐target interactions—insights that are essential for the rational design of smart and responsive delivery systems. Potential emerging technologies and their prospective applications are also discussed.

### 3.1 Supramolecular Organization of Drug Nanocarriers

The physicochemical properties of drug nanocarriers significantly influence many key parameters, including their payload delivery, cellular uptake, intra‐cellular trafficking, release efficiency, and nanotoxicity.^[^ ^66^, ^204^, ^205^, ^206^, ^207^, ^208^ ^]^ Here, we focus mainly on the applications of computational approaches in the study of several key physicochemical properties of nanocarriers: (i) the morphology, including size and shape; (ii) the mechanical properties, such as stiffness; (iii) the surface properties and functionalization, and (iv) the spatial distribution of their components/building blocks. Furthermore, we discuss the influences of intra‐ and intermolecular interactions within polymer‐based nanocarriers, which are responsible for their self‐assembly, colloidal stability, and drug delivery capabilities.

#### 3.1.1 Morphology of Nanocarriers

The size and shape of nanocarriers are crucial factors influencing their ability to load drugs, the selection of the administration route, and their in vivo fate. It has been reported that, while nanoparticles smaller than 200 nm in diameter are safely intravenously injectable, the optimal nanoparticle size for penetrating tumors should be in the range of 10–100 nm.^[^ ^209^ ^]^ They can penetrate and cross various tissues, enabling the drug to be delivered to targeted cells without the risk of embolism. However, when their size exceeds 500 nm, they can hardly circulate in blood vessels or penetrate tissues.^[^ ^210^ ^]^ The shape of nanocarriers also plays an important role in drug delivery performance. It has been reported that non‐spherical nanocarriers—such as worm‐like, elliptical/elongated particles—exhibit significantly different solvent‐accessible surface areas (SASA), hydrodynamic radii, and solution viscosities compared to spherical counterparts. These anisotropic geometries tend to promote alignment and tumbling behaviors during circulation, particularly when passing through biological filtration organs, whereas spherical particles typically exhibit more predictable flow dynamics. Moreover, variations in nanocarrier surface curvature also influences the extent to which particles conform to the curvature of cellular membranes.^[^ ^57^ ^]^ These factors collectively contribute to distinct blood circulation and cellular uptake efficiencies. Another study reported that filamentous polymer micelles exhibited significantly prolonged circulation properties in rodents, with a circulation time tenfold longer than that of their spherical counterparts and short filomicelles.^[^ ^211^ ^]^ This difference can be attributed to the fact that the longer filaments are more extended by the flow, thus avoiding cellular uptake, conversely to their spherical counterparts.

The size and shape of nanocarriers can be controlled by various experimental factors, including the synthesis strategy, the incorporation of surfactant, the solvent effects, the nature of the polymer and its concentration, the temperature, and pH, etc.^[^ ^212^, ^213^ ^]^ To anticipate or rationalize how these effects impact nanoparticle formation, and to gain a more comprehensive understanding of how nanoparticle size and shape influence the design of drug delivery systems, more and more studies are using computational techniques to systematically investigate their intrinsic relationship at the molecular level.

**Figure 3:**
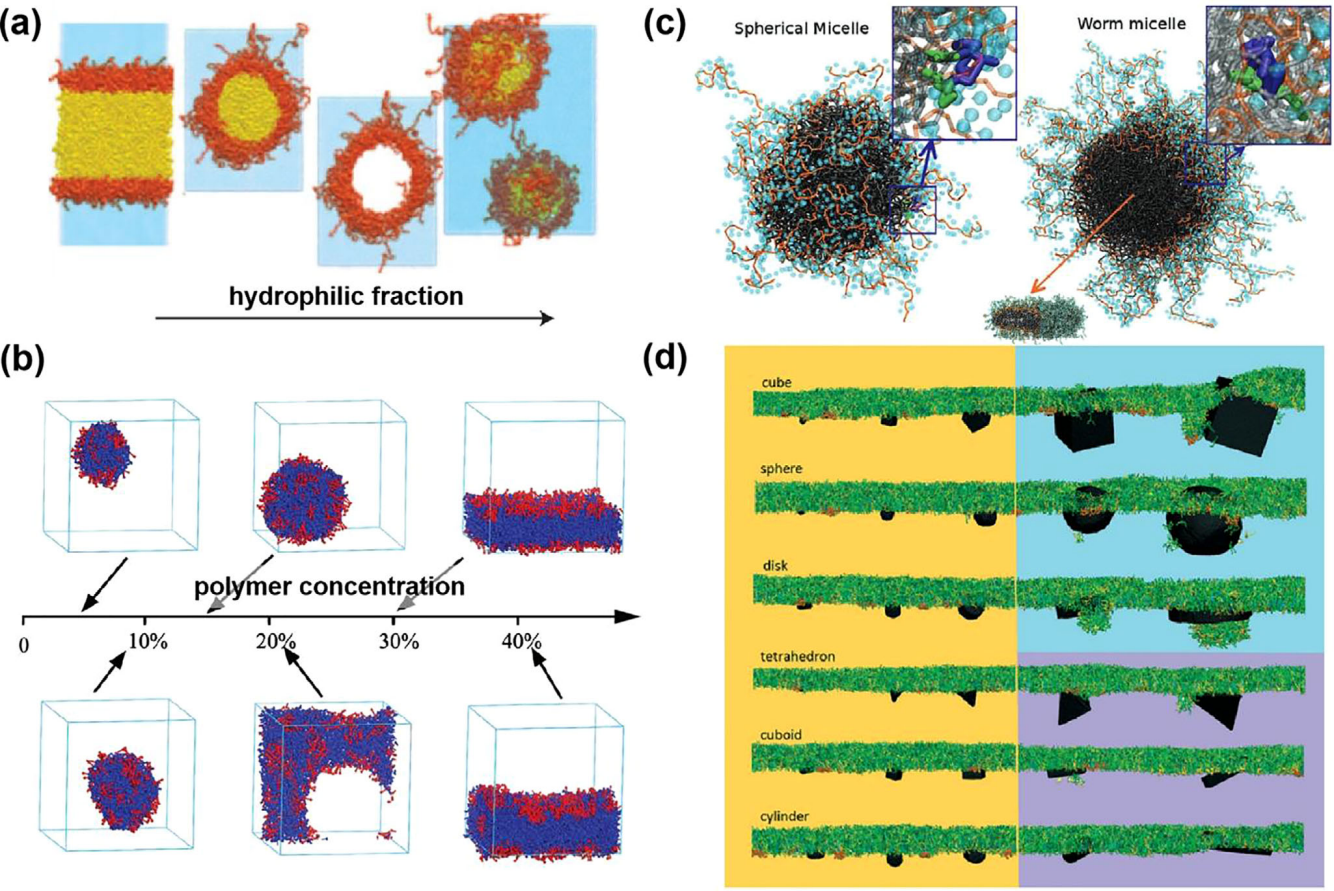
a) Snapshots of PEO‐*b*‐PEE diblock copolymer micelle morphologies as a function of hydrophilic fraction (*f_phil_*, w/w) obtained by CG MD simulations. Reproduced with permission.^[^ ^214^ ^]^ Copyright 2004, Springer Nature. b) Morphological changes in PLLA_8_‐PC‐PLLA_8_ self‐assembly with increasing polymer concentration. Reproduced with permission.^[^ ^145^ ^]^ Copyright 2011, American Scientific Publishers. c) Morphologies of spherical micelle and worm copolymer micelles (PEG_2000_‐*b*‐PCL_5000_) with Ptx constrained near the interface. PEG (in orange) is shown with its hydration layer of CG water. Ptx is represented in blue‐green. The orange arrow indicates the symmetry axis of the worm micelle. Reproduced with permission.^[^ ^217^ ^]^ Copyright 2011, Wiley‐VCH Verlag GmbH. d) Size‐ and shape‐dependent translocation of hydrophilic silica nanoparticles across a PS layer obtained by CG MD simulations. Reproduced with permission.^[^ ^218^ ^]^ Copyright 2018, the Royal Society of Chemistry.

Among the simulation techniques outlined in the previous section, CG MD, and DPD have emerged as preferred methods for capturing self‐assembly behavior, owing to their ability to access extended spatial and temporal scales. Notably, numerous studies have demonstrated that the ratio of hydrophilic to hydrophobic components plays a critical role in dictating the morphology and size of polymer nanocarriers. For example, CG MD simulations were used to study the self‐assembly process and shapes transformation of poly(ethyl ethylene)‐*block*‐poly(ethylene oxide) (PEE‐*b*‐PEO) amphiphilic diblock copolymer micelles. Simulations revealed that the shape of these micelles changed from bilayer, cylindrical or worm‐like to spherical as the proportion of hydrophilic fractions (*f_phil_*, w/w) increased (**Figure** **3**a).^[^ ^214^ ^]^ In another example, the influence of the chain length and concentration of poly(L‐lactide)‐*block*‐phosphorylcholine‐*block*‐poly(L‐lactide) (PLA‐*b*‐PC‐*b*‐PLA) triblock copolymers on their self‐assembly was investigated by a DPD approach.^[^ ^145^ ^]^ The authors showed that decreasing the polymer chain length gave bilayer structures, while increasing polymer concentration favored the formation of spherical, cylindrical, and then lamellar nanoparticles (Figure 3b). Moreover, subtle variations in the molecular architecture of the polymer have also been reported to exert a pronounced influence on nanocarrier morphology. For example, Jiang and co‐workers, through multiple long‐timescale self‐assembly simulations at both AA and CG resolutions, demonstrated that subtle modifications to the head‐group architecture of telodendrimers—specifically G1 PEG^5K^CA_8_, G2 PEG^5K^RH_4_CA_4_, and G3 PEG^5K^CA_4_‐L‐RH_4_—resulted in three distinct morphologies: aspherical nanoparticles, wormlike micelles, and elongated wormlike nanocarriers.^[^ ^215^ ^]^ These architectural variations further dictated unique molecular packing arrangements and produced markedly different morphological outcomes upon doxorubicin (Dox) loading.

The chemical nature of the drug and its loading configuration can substantially influence the nanocarrier's structural characteristics. In one representative study, a multiscale model of poly‐*γ*‐glutamyl‐glutamate‐paclitaxel (PGG‐Ptx) polymer prodrugs was constructed by first employing AA MD and circular dichroism (CD) spectroscopy to characterize the secondary structure of PGG‐Ptx in solution. This atomistic insight then served as the basis for constructing a CG model that preserved essential structural features while reducing complexity. Using this multiscale framework, the effects of Ptx loading and its spatial arrangement along the polymer backbone on overall chain size and shape were systematically studied.^[^ ^216^ ^]^ Simulation results indicated that individual PGG‐Ptx polymer prodrug chains prefer to form coil (linear) rather than globular (round) shapes, facilitating longer circulation times and greater drug accumulations in tumor endothelial walls. The influence of drug loading extends beyond individual polymer chains to the supramolecular organization of nanocarriers. For instance, Loverde and coworkers studied the correlation between the Ptx loading and the morphology of poly(ethylene glycol)‐*b*‐poly(caprolactone) (PEG‐*b*‐PCL) micelles using rational CG MD simulations.^[^ ^217^ ^]^ Their findings indicated that worm‐like micelles exhibited nearly double the drug‐loading capacity of spherical micelles. Moreover, in spherical micelles, Ptx molecules preferentially localized near the inner interface of the micellar corona, which is thought to be responsible of the “burst release” phenomenon (Figure 3c).

Beyond the aforementioned cases, increasing research efforts have been directed toward understanding how the shape and size of nanoparticles influence their translocation across lipid bilayers by computational methods.^[^ ^219^, ^220^ ^]^ Similarly, CG models are often favored for these studies, as they enhance simulation efficiency by reducing the resolution of the lipid and polymer representation while preserving their chemical specificity.^[^ ^221^, ^222^, ^223^ ^]^ For example, CG MD simulations were used to demonstrate that hydrophilic silica nanoparticles of small size (<5 nm) can quickly penetrate the pulmonary surfactant (PS) layer, being barely affected by their shape.^[^ ^218^ ^]^ In contrast, the translocation of larger nanoparticles was more affected by their shape (Figure 3d), among which nanoparticles with sharp corners or needle‐like shapes more easily crossed the PS layer. This trend was attributed to the absorption of PS on the nanoparticle surface during the translocation process, as well as to the disruption of the PS arrangement around the sharp edges of the nanoparticles, resulting in the disruption of the PS layer. Overall, while computational methods have provided valuable details into the factors governing nanoparticle morphology and its influence on their dynamic transfer process across bilayers, current limitations in computational resources often necessitate the use of nanoparticle sizes smaller than those employed in experimental studies. As such, caution is advised when interpreting these results.

#### 3.1.2 Stiffness of Nanocarriers

The stiffness of nanocarriers is another important parameter which impacts their blood circulation, cellular uptake, and drug delivery. Recent reports have highlighted the use of nanoparticles and micelles with specific stiffness for biomedical applications.^[^ ^204^, ^224^, ^225^, ^226^ ^]^ For example, unlike rigid nanoparticles (e.g., gold‐based nanoparticles or carbon‐based nanotubes), deformable nanoparticles (e.g., micelles) exhibited lateral motion in the bloodstream due to the hydrodynamic lifting forces induced by deformation. Consequently, deformable nanoparticles are able to move away from the vein walls to present a prolonged circulation in the bloodstream.^[^ ^227^ ^]^ In addition, particle stiffness also plays a crucial role in mediating cellular internalization.^[^ ^228^, ^229^ ^]^ For example, the uptake efficiency of hyaluronic acid methacrylate (HAM)‐based capsules by HeLa cells has been shown to be inversely correlated with their stiffness—that is, softer capsules exhibit stronger interactions with the cell membrane, leading to more efficient internalization.^[^ ^230^ ^]^

**Figure 4:**
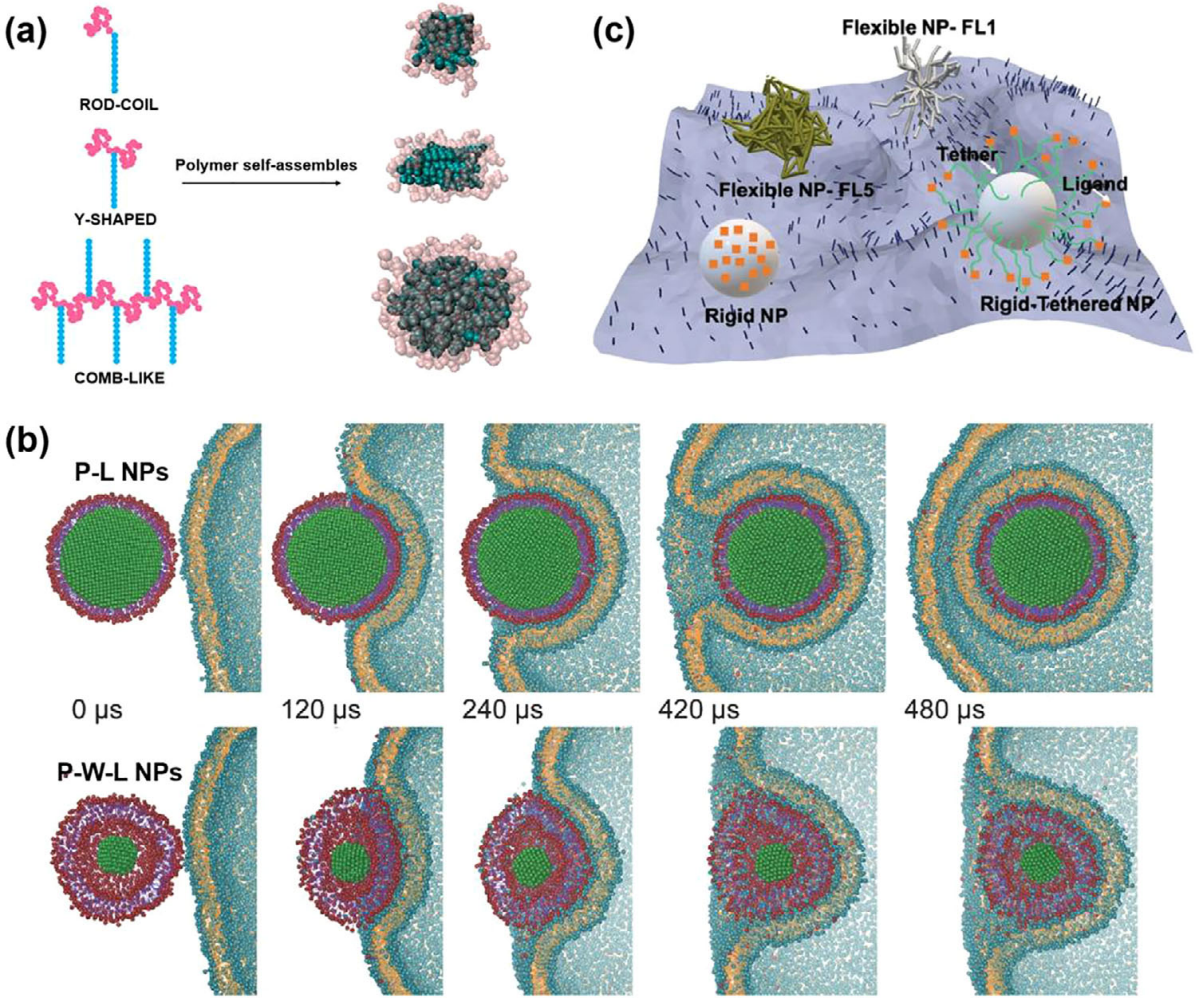
a) Schematic representation of micelles formed by rod‐coil, Y‐shaped, and comb‐like copolymers. Reproduced with permission.^[^ ^233^ ^]^ Copyright 2017, American Chemical Society. b) Screenshot of cell membrane deformation during cellular uptake of rigid P‐L NPs and flexible P‐W‐L NPs by MD simulations. Reproduced with permission.^[^ ^237^ ^]^ Copyright 2014, Wiley‐VCH Verlag GmbH. c) Illustration of various types of nanoparticles exhibiting different stiffness interacting with an undulating cell membrane, performed by MC simulations. Reproduced with permission.^[^ ^239^ ^]^ Copyright 2021, Wiley‐VCH GmbH.

Recently, computational techniques have been increasingly employed to elucidate the role of stiffness and its modulation through structural parameters. These investigations were broadly categorized into three major aspects: (i) the effect of polymer rigidity on micellization: It was reported that rigid polymer chains exhibited a lower critical micelle concentration (CMC) and formed larger and more well‐defined micelles compared to flexible chains.^[^ ^231^ ^]^ However, as rigidity increased beyond a certain threshold, its influence on micellization became less pronounced. (ii) The effect of chain rigidity on micelle morphology and stability: It was also observed that solutions containing flexible and rigid molecules with similar CMCs can exhibit substantial differences in micelle size and shape.^[^ ^232^ ^]^ In another example, the self‐assembly of Y‐shaped (insoluble rigid block and two flexible soluble arms) and comb‐like (soluble flexible backbone with insoluble rigid side chains) copolymers was investigated by DPD simulations (**Figure** **4**a).^[^ ^233^ ^]^ It was found that bulky comb‐like macromolecules with flexible main chains and rod‐like side chains were more likely to form near‐spherical soft micelles. Additionally, the impact of chain rigidity on the CMC and micelle morphology of model surfactants was investigated by using grand canonical MC simulations.^[^ ^234^ ^]^ By tuning the rigidity of the entire chain, it was demonstrated that fluorocarbon surfactants—with intrinsically higher conformational stiffness—tend to assemble into micelles of larger inner micellar volume, in contrast to their more flexible hydrocarbon analogs. (iii) The impact of nanoparticle stiffness on their biological fate: Recent computational efforts have also explored how nanoparticle stiffness modulates interactions with biological interfaces, including cellular uptake, membrane penetration, and targeting efficiency.^[^ ^235^, ^236^ ^]^ For instance, CG models have been used to study potential differences in cellular uptake of core‐shell PLGA‐lipid (P‐L) nanoparticles with tunable stiffnesses.^[^ ^237^ ^]^ Flexible P‐W‐L nanoparticles, with a water layer between the PLGA core and the lipid shell, were found to be energetically unfavorable for cell uptake compared to rigid P‐L nanoparticles (Figure 4b). Similarly, CG simulations of nanoparticles made of a lysozyme‐rich core coated with a dextran shell demonstrated that ultra‐soft polymer nanoparticles led to a significantly increase in targeting due to a multivalent binding effect.^[^ ^238^ ^]^ In another report, the interactions and dynamics of nanoparticles of different stiffness (e.g., core‐corona flexible nanoparticles, rigid nanoparticles, or rigid‐tethered nanoparticles) with membranes via biophysical modeling were investigated using MC simulations (Figure 4c).^[^ ^239^ ^]^ It was shown that soft and flexible nanoparticles can maximize multivalent interactions with the membrane surface, making them superior to rigid nanoparticles in terms of cell binding affinity.

While CG and DPD models dominate in the aforementioned fields due to their scalability and computational efficiency, it should be noted that AA simulations remain essential for probing fine‐grained mechanical properties—such as chain elasticity, twisting stiffness, and bending persistence length—which are particularly relevant in systems like DNA.^[^ ^240^ ^]^ Overall, the introduction of computational studies not only helped to understand the relationship between the influence of polymer/lipid nature on the variation of nanoparticle stiffness, but also to gain insight into the association between nanoparticle stiffness and their cellular binding or internalization process, which can be a great assistance in designing flexible polymer nanoparticles for drug delivery applications.

#### 3.1.3 Surface Properties of Nanocarriers

**Figure 5:**
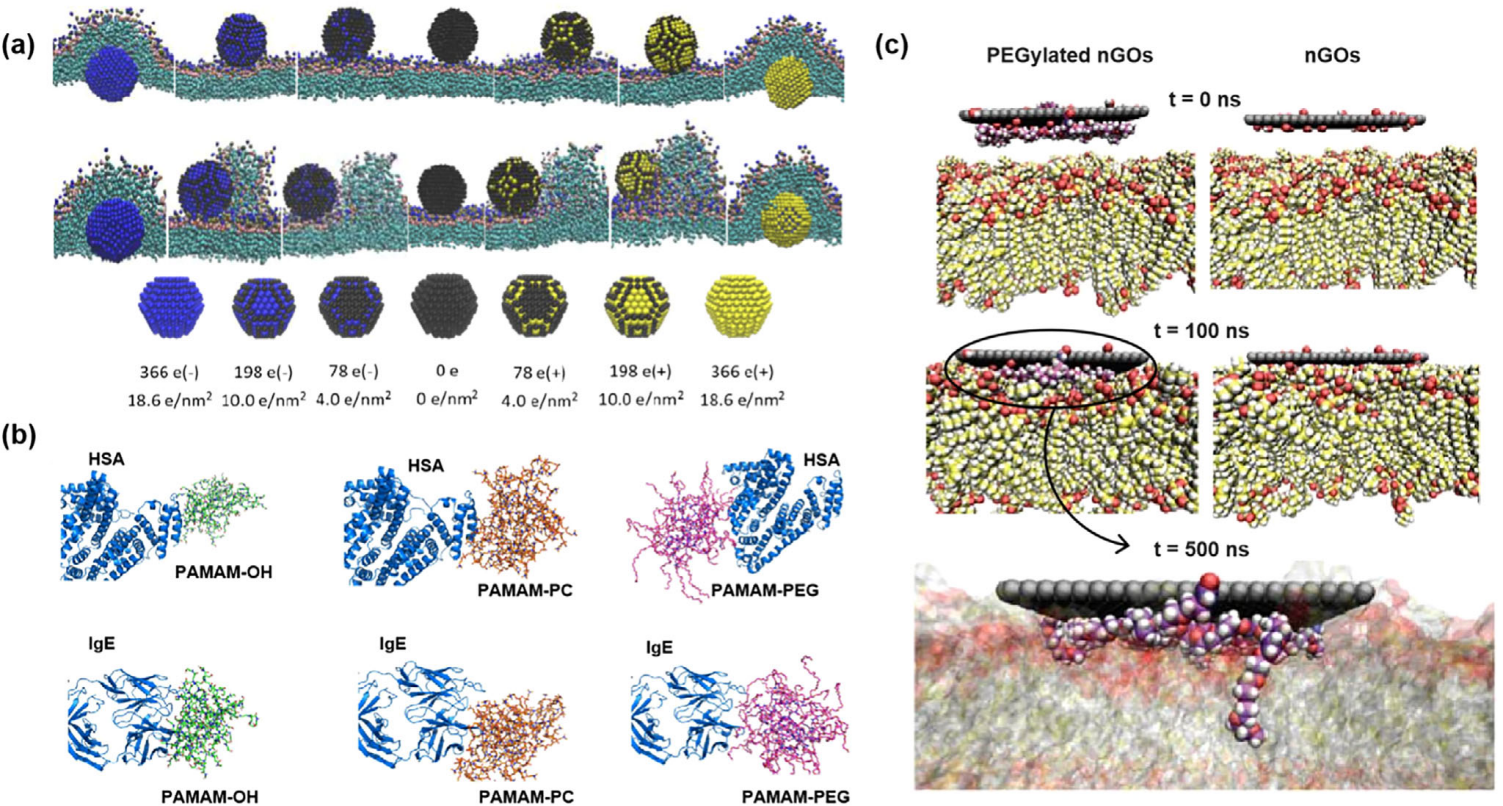
a) Illustration of the transfer process of hydrophilic nanoparticles with different surface charge densities (positive charge and negative charge) across the pulmonary surfactant monolayer in an expanded (top) or compressed state (bottom), performed by CG MD simulations. Reproduced with permission.^[^ ^247^ ^]^ Copyright 2017, Taylor & Francis. b) Snapshots of OH‐, PC‐, and PEG‐modified PAMAM dendrimer nanoparticles binding with HSA (top) and IgE (bottom) from DMD simulations. Reproduced with permission.^[^ ^251^ ^]^ Copyright 2018, American Chemical Society. c) Representative simulation snapshots of the membrane adsorption process of PEGylated nGO and bare nGO. Illustration of PEG chains desorption from the nGO surface facilitates the insertion of PEG anchors into the lipid bilayer. Reproduced with permission.^[^ ^259^ ^]^ Copyright 2017, Springer Nature.

The surface properties of polymer nanoparticles are also of crucial importance as they govern their biocompatibility, as well as their cellular uptake and targeting properties, which are key parameters for drug delivery applications.^[^ ^241^, ^242^ ^]^ Among these, surface charge is particularly critical due to its strong influence on nanoparticle‐cell membrane interactions. In general, nanoparticles with a positive surface charge exhibit more efficient cell internalization, given that the cell surface is rather negatively charged.^[^ ^243^ ^]^ This was notably illustrated with PLGA‐based nanoparticles, the surface charge of which could be adjusted from negative to positive by modifying the PLGA carboxylic acid end groups with chitosan, resulting in an increased affinity of the nanoparticles for cancer cells.^[^ ^244^ ^]^ This surface charge‐dependent property was investigated in two other studies where pH‐sensitive zwitterionic polymer nanocarriers have been found to remain neutral at physiological pH, thus reducing their interactions with the immune system. When the pH was decreased, the nanocarriers became positively charged, resulting in greater permeability across negatively charged acidic cancer cell membranes.^[^ ^245^, ^246^ ^]^ The introduction of simulation techniques has also made it possible to overcome certain experimental limitations and offered a rational explanation for understanding the influence of nanoparticle surface charge on their cell uptake process and the interaction mechanisms. For example, the translocation mechanism of nanoparticles with varying surface charge densities across a surfactant monolayer was investigated using CG MD simulations.^[^ ^247^ ^]^ The results showed that the nanoparticles with the highest surface charge densities exhibited the lowest cellular internalization, which was attributed to their interactions with the pulmonary surfactant monolayer, causing a significant structural deformation that altered the normal phase transition dynamics of the monolayer during the translocation process (**Figure** **5**a). In another study, the interaction dynamics of neutral or protonated Dox‐loaded PEGylated hyperbranched polyester (HBP) nanoparticles with a negatively charged lipid bilayer were studied using AA simulations.^[^ ^248^ ^]^ Interestingly, the protonated drugs exhibited a tendency to accumulate in proximity to the nanoparticle surface, in contrast to their neutral counterparts. Such a phenomenon can be attributed to the strong induction of the lipid bilayer on the former, which subsequently facilitated the diffusion of the positively charged drugs from the nanoparticle toward the membrane.

Beyond surface charge, surface functionalization also plays a pivotal role in tuning polymer nanoparticle performance. Hydrophilic polymers, such as PEG and polysaccharides such as chitosan, are commonly grafted onto nanoparticle surfaces to improve colloidal stability, prolong circulation time, and enhance biocompatibility.^[^ ^249^, ^250^ ^]^ To achieve active targeting, their surface‐functionalization with homing ligands (e.g., vitamins, peptides, proteins, antibodies, aptamers) is also widely investigated.^[^ ^5^ ^]^ Computational techniques have increasingly been introduced to better understand the effect of polymer nanoparticle surface modifications on their biological applications. For instance, the influence of surface modification of polyamidoamine (PAMAM) dendrimer nanoparticles with hydrophilic compounds (neutral PC, PEG, or hydroxyls (OH)) on their interaction with serum proteins has been studied by discrete molecular dynamics (DMD) simulations.^[^ ^251^ ^]^ The result demonstrated that the surface modification of PAMAM dendrimers with above mentioned neutral OH, PC, and PEG significantly reduced their binding affinity with immunoglobulin (Ig) proteins and human serum albumin (HSA), in comparison with the modification with positively or negatively charged PAMAM nanoparticles (Figure 5b). It was reported in another example that the surface modification of PAMAM dendrimer nanoparticles with amine, acetyl, or carboxyl terminal groups resulted in enhanced chalcone loading and stability at physiological environments using AA MD simulations.^[^ ^252^ ^]^

Recently, bio‐inspired hybrid materials—such as carbon‐based nanomaterials functionalized with polymers—have shown considerable promise in bridging high drug‐loading capacity with biocompatibility. When functionalized with PEG, chitosan, or PLGA, carbon nanotubes (CNTs), graphene oxide (GO), and silica nanoparticles exhibit improved solubility, reduced immunogenicity, and extended circulation stability.^[^ ^253^, ^254^, ^255^ ^]^ Moreover, such surface functionalization enables the development of stimuli‐responsive drug delivery systems—particularly pH‐sensitive nanocarriers such as 5‐fluorouracil loaded chitosan/agarose/graphene oxide nanocomposites (CS/AG/GO/5‐FU),^[^ ^256^ ^]^ chitosan coated quercetin‐loaded mesoporous silica^[^ ^255^ ^]^ that can selectively release therapeutic agents in acidic tumor microenvironments, thereby enhancing targeting specificity and therapeutic efficacy. Molecular simulation techniques have increasingly contributed to the mechanistic understanding and design of polymer‐modified carbon nanomaterials in drug delivery.^[^ ^257^, ^258^ ^]^ For instance, MD simulations were employed to show that PEGylated nanoscale GO (nGO) nanosheets preferentially adsorb onto and partially insert into cell membranes, thereby triggering strong cytokine responses (Figure 5c).^[^ ^259^ ^]^ In another study, MD simulations were conducted on three silica‐filled polymer nanocomposites composed of CS, PEG, and PLA for chlorambucil (CB) delivery, finding that CS‐based systems provided the most effective transport and diffusion properties among the tested platforms.^[^ ^260^ ^]^ Additionally, MC simulations were utilized to assess the interactions between imatinib (IMA) and CNTs.^[^ ^261^ ^]^ The results revealed that electrostatic interactions were the dominant force in binding, and solvation free energy calculations showed that carboxyl and hydroxyl functionalized CNTs substantially enhanced the aqueous solubility of IMA, suggesting potential improvements in its bioavailability. Although a full account of these studies is beyond the scope of this review, they collectively underscore the utility of computational methods in guiding the rational design of polymer‐nanomaterial hybrid systems for advanced drug delivery applications.

#### 3.1.4 Drug Distribution in Nanocarriers

**Figure 6:**
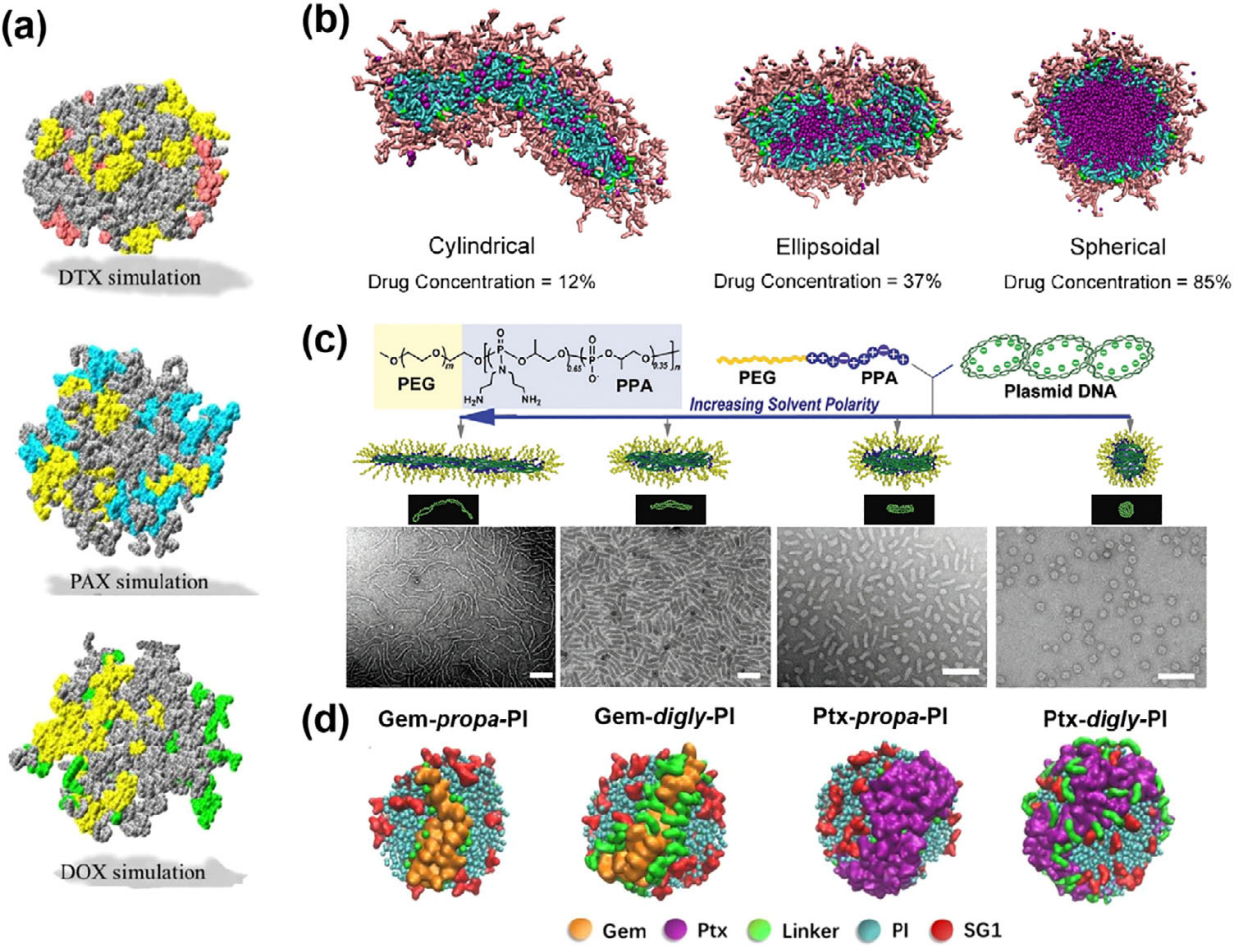
a) Spatial distribution in CG model of three different drugs (Dtx, Ptx, and Dox) after their physical encapsulation into PLGA‐*b*‐PEG/PLGA‐*b*‐PEG‐Rf polymer nanoparticles. Dtx (pink), Pax (blue), Dox (green), PLGA‐*b*‐PEG (grey), and PLGA‐*b*‐PEG‐RF (yellow). Reproduced with permission.^[^ ^262^ ^]^ Copyright 2022, Springer Nature. b) Morphology transition of drug‐PGMA‐*g*‐PEG/PLA aggregates at different Dox concentrations in DPD model. Dox (purple), PEG (pink), PLA (blue), and PGMA (green). Reproduced with permission.^[^ ^263^ ^]^ Copyright 2022, American Chemical Society. (c) Morphologies in CG model (top) and TEM images (bottom) of PEG_10K_‐*b*‐PPA_4K_/DNA micelles in solvents of different polarities (deionized water and DMF/water mixtures of 3:7 (v/v), 5:5 (v/v) and 7:3 (v/v), from left to right). PEG (yellow), PPA (purple), and plasmid DNA (green). Reproduced with permission.^[^ ^267^ ^]^ Copyright 2012, Wiley‐VCH Verlag GmbH. (d) Drug and polymer distribution in Gem‐ and Ptx‐polymer prodrug nanoparticles in CG model. Reproduced with permission.^[^ ^268^ ^]^ Copyright 2022, American Chemical Society.

The self‐assembly of polymer nanocarriers is inherently a dynamic process that governs the evolution of their morphology and internal organization. In the context of drug delivery systems, the spatial distribution of individual components—such as polymer segments, encapsulated drugs, and functional moieties—plays a critical role in determining delivery efficiency. In particular, the localization of drug molecules within the nanocarrier matrix is a key determinant of release kinetics and, ultimately, therapeutic efficacy. However, the inherent limitations of experimental techniques make this characterization rather difficult. Drug localization in nanocarriers depends on their physicochemical parameters (e.g., solubility in water), their compatibility with the polymer matrix, and, in the case of polymer prodrugs, their positioning on the polymer backbone. In this context, large‐scale simulation models have provided researchers with a robust tool for accessing this information and better predict the behavior of drug‐loaded polymer nanocarriers. For example, the location of different anticancer drugs (docetaxel (Dtx), Ptx, and Dox) encapsulated in Riboflavin (Rf)‐conjugated PLGA‐*b*‐PEG/PLGA‐*b*‐PEG‐Rf polymer nanoparticles has been investigated using both AA and CG simulations.^[^ ^262^ ^]^ The results indicated that the drugs were entrapped in the hydrophobic PLGA core, as seen on the MD snapshots (**Figure** **6**a). In contrast, the more hydrophilic Rf and PEG were located at the surface of the nanoparticles, allowing the Rf moieties to remain sufficiently accessible, facilitating targeting to cancer cell receptors.

Molecular simulation methods were also successfully used to study the impact of drug loading on the supramolecular organization of nanocarriers and offer valuable insights into optimal drug‐to‐polymer ratios for experimental formulation design. For instance, the morphology of poly(glycidyl methacrylate)‐*graft*‐poly(ethylene glycol)/polylactide (PGMA‐*g*‐PEG/PLA) polymer aggregates with different amounts of Dox (0.12, 0.37, and 0.85, defined as the ratio between the total number of drug beads in the simulation box and the total number of beads of the bottlebrush) were studied using DPD simulations.^[^ ^263^ ^]^ The results showed that for a drug loading of 12%, the Dox molecules were nearly entirely entrapped in the core of the cylindrical aggregate, and that the PEG chains were well distributed on their surface, while the hydrophobic PLA was distributed inside the core. As the drug loading increases, more and more Dox molecules begin to accumulate in the center of the aggregate, forming a droplet, while PGMA‐*g*‐PEG/PLA wraps around the droplet to form a spherical aggregate (Figure 6b). Interestingly, very similar results were observed with PEG‐*b*‐PLA‐*b*‐PEG micelles.^[^ ^264^ ^]^ The distribution of Dox in three‐layer core‐mesosphere‐shell micelles made of poly(*ε*‐caprolactone)‐*b*‐poly(2‐(diethylamino)ethyl methacrylate)‐*b*‐poly(poly(ethylene glycol) methyl ether methacrylate) (4AS‐PCL‐*b*‐PDEAEMA‐*b*‐PPEGMA) was also investigated.^[^ ^149^ ^]^ Their DPD simulations showed that the Dox molecules were distributed within the core and at the interface between the core and the mesosphere. As drug loading increased, the distribution of Dox became more concentrated within the PCL shell, resulting in some openings in the shell, potentially leading to unwanted drug release.

The drug encapsulation behavior of telodendrimers has also been studied using multiscale computational approaches. For example, Jiang and co‐workers examined drug–telodendrimer micelles composed of poly(ethylene glycol)‐*b*‐dendritic oligo(cholic acid) block copolymers as the polymer scaffold.^[^ ^265^ ^]^ The CG simulations revealed that Ptx molecules were predominantly localized within the hydrophobic core of the micelles at a low drug loading of 5% and 17%. However, as the drug loading increased to 25% and 36%, PTX distribution extended toward the micelle periphery, leading to partial exposure of the drug at the surface. This redistribution compromised the ability of telodendrimers to effectively shield the PTX core, allowing water penetration into the micelle interior, which destabilized the assembly and resulted in a leaky structure prone to burst release. In addition, the effect of solvent polarity on the supramolecular organization of nanocarriers has also been studied by molecular simulation methods.^[^ ^195^, ^266^ ^]^ A recent study by Jiang and co‐workers showed that the morphology of plasmid DNA‐PEG‐*b*‐polyphosphoramidate copolymer nanoparticles (PEG_10K_‐*b*‐PPA_4K_/DNA) was strongly influenced by solvent polarity.^[^ ^267^ ^]^ By varying the solvent polarity, from deionized water to DMF/water mixtures of 3:7 (v/v), 5:5 (v/v), and 7:3 (v/v), the condensed plasmid DNA in the nanoparticle core underwent conformational changes; from worm‐like shape to a folded rod‐like morphology and then to a compact spherical structure, in good agreement with experimental evidences (Figure 6c).

As far as polymer prodrugs are concerned, only a limited number of studies have used computational simulations to provide complete localization of drug, linker, and polymer in self‐assembled prodrug nanoparticles. In this area, CG MD has proved effective in revealing the supramolecular organization of Gemcitabine (Gem) and Ptx‐polyisoprene prodrug nanoparticles bearing various polymer‐drug linkers.^[^ ^268^ ^]^ The simulation results showed that the hydrophilic Gem molecules were mainly located at the periphery of the nanoparticles, whereas the hydrophobic Ptx molecules were more deeply embedded in the core of the nanoparticles, their polar groups being however, visible on the surface (Figure 6d). Simulations of methoxy poly(ethylene glycol)‐*b*‐poly(*ε*‐caprolactone‐*co*‐6‐carboxylic‐*ε‐*caprolactone‐*g*‐5‐aminolevulinic acid methyl ester) (mPEG‐*b*‐P(CL‐*co*‐mACCL)) polymer prodrug micelles with variable grafting ratios and physically encapsulated 5‐aminolevulinic acid (m‐ALA) were performed using a DPD model.^[^ ^269^ ^]^ The results indicated that m‐ALA was relatively uniformly distributed in the entire box when the grafting ratio was 0%. However, when the grafting rate was increased, both the encapsulated and grafted m‐ALA clustered together to form aggregates at the core of the micelles due to the increased compatibility between the polymer prodrugs and drugs.

#### 3.1.5 Molecular Mechanisms Governing Polymer Self‐Assembly

**Figure 7:**
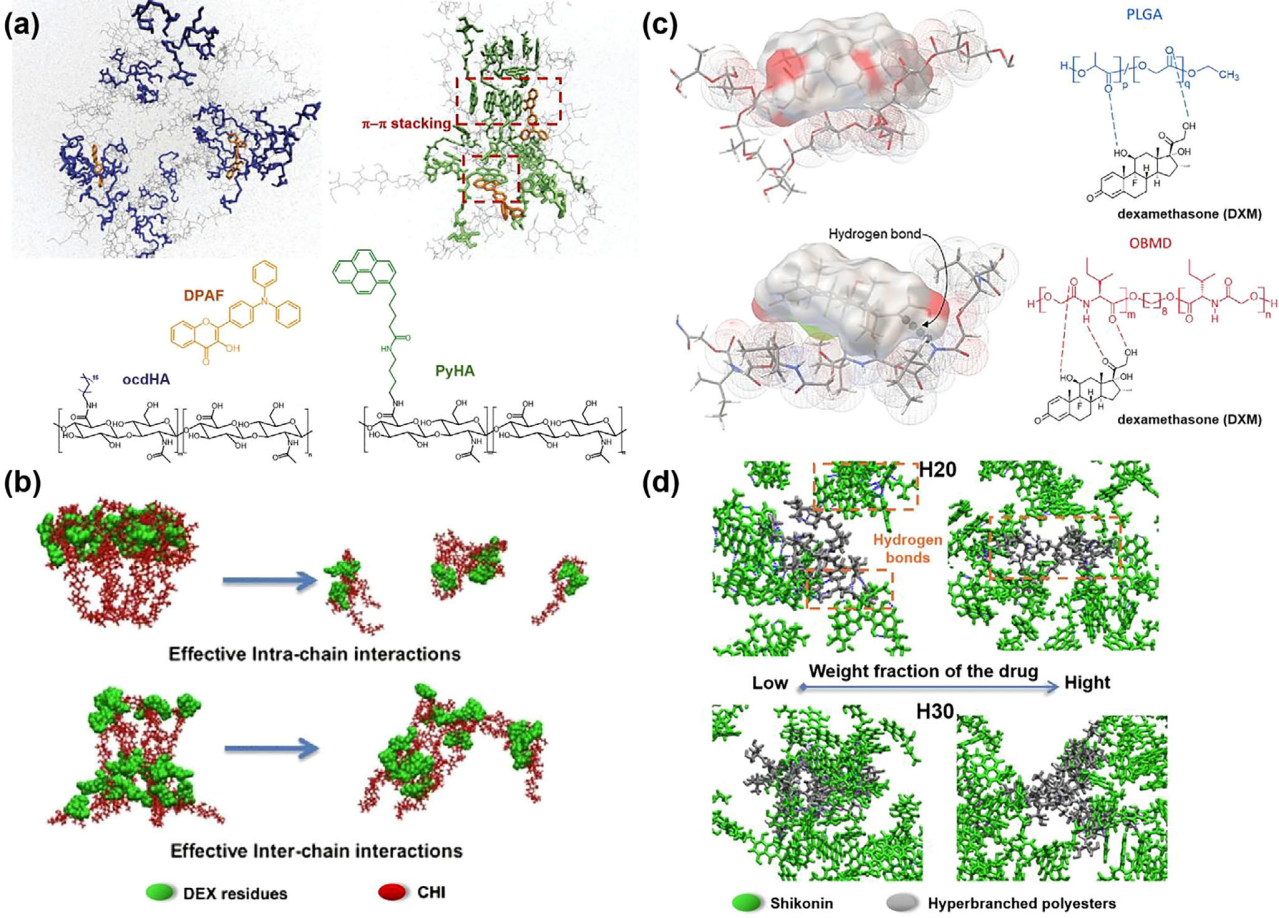
a) Snapshots of two molecules of dye DPAF interactions with four molecules of 10kDda ocdHA (left) or PyHA (right) in AA MD simulation. HA backbone is colored light gray. The multilayer π–π stacking are circled in red for clarity. Reproduced with permission.^[^ ^271^ ^]^ Copyright 2018, Royal Society of Chemistry. b) The balance between intra‐ and inter‐chain interactions affects the self‐assembly behavior of CHI‐DEXs. Reproduced with permission.^[^ ^272^ ^]^ Copyright 2015, Springer Nature. c) DEX interactions with PLGA (top) and OBMD (bottom) in MD and docking studies. Their chemical structures and H‐bond interactions are shown on the right for clarity. Reproduced with permission.^[^ ^273^ ^]^ Copyright 2019, Elsevier. d) Snapshots of hyperbranched polyesters H20 (top) and H30 (bottom) interacting with different concentrations of shikonin. Short dotted lines indicate hydrogen bonds, which are circled in orange. Reproduced with permission.^[^ ^274^ ^]^ Copyright 2011, the Royal Society of Chemistry.

The balance between polymer‐polymer, polymer‐payload/drug, drug‐drug, and solute‐solvent interactions determines nanoparticle formation, stability, morphology, drug loading, spatial drug distribution, and drug release kinetics.^[^ ^65^, ^270^ ^]^ Computational biophysical studies can be very useful in assessing the role of the hydrophilic/hydrophobic nature of drugs and polymers on nanoparticle physicochemical properties and encapsulation strategies. Among them, AA MD simulations are widely used to capture atomistic details of such interactions, including hydrogen bonds and packing effects. For example, interactions between amphiphilic hyaluronic acid (HA) derivatives bearing aliphatic octadecyl (ocdHA) or aromatic pyrene butanamide (PyHA) side substituents and dye (DPAF) molecules have been studied by AA MD simulations.^[^ ^271^ ^]^ The results showed that ocdHA hydrocarbon chains tended to wrap DPAF molecules in the hydrophobic pocket to form a less compact aggregate and more extensive hydrophobic domains, while aromatic moieties allowed for greater interactions with DPAF molecules and the surrounding solvent (**Figure** **7**a).

AA MD simulations were also employed to study how the grafting of hydrophobic salicylic acid (SA) on chitosan oligosaccharide (COS) promoted the aggregation of these polymers into core‐shell organized micelles, as well as the encapsulation of Ptx.^[^ ^169^ ^]^ Although hydrophobic interactions are the main driving force behind COS‐SA self‐assembly, MD simulations revealed that intermolecular hydrogen bonds contributed significantly to the aggregation process and nanoparticle stabilization. It is interesting to note that the number of grafted SA per COS chain determined the size of the hydrophobic core and, therefore, the drug loading. In another study, the molecular interactions governing the self‐assembly of amphiphilic chitosan (Chi) as a function of grafted hydrophobic dexamethasone (DEX) were elucidated by using AA MD simulations.^[^ ^272^ ^]^ It was shown that increasing the hydrophobicity of Chi with DEX moieties did not necessarily promote the self‐assembly into micelles with lower critical aggregation concentration, smaller size, and lower zeta potential. MD simulations have demonstrated that the balance between intra‐ and intermolecular interactions governed the equilibrium between compact and extended conformations of the single chains, which, in turn, determined the self‐assembly propensity of Chi‐DEX (Figure 7b). Computational studies on the interactions between drugs and single polymer chains can also be of great interest to provide useful information about non‐covalent drug‐polymer interactions, and help to rationalize and optimize the drug loading of polymer‐based nanocarriers. Molecular docking and MD studies have shown that the oligodepsipeptide oligo[3‐(*S*)‐*sec*‐butylmorpholine‐2,5‐dione]diol (OBMD) with amide bonds in the backbone can act as H‐bond donors to interact with the carbonyl group of DEX, enabling enhanced drug loading compared to the case for PLGA (Figure 7c).^[^ ^273^ ^]^ In addition, AA MD simulations were used to study the interactions between: (i) poly(ethylene oxide)‐*b*‐poly(caprolactone) (PEO‐*b*‐PCL) chains and drugs containing either hydrogen bond donors and acceptors (cucurbitacin B and cucurbitacin I), or only hydrogen bond acceptors (fenofibrate and nimodipine),^[^ ^171^ ^]^ and (ii) hyperbranched polyesters of the second and third (pseudo)generations (named H20 and H30) with shikonin as the drug (Figure 7d).^[^ ^274^ ^]^ Both studies indicated that hydrogen bonding was the main driving force behind drug‐polymer associations and determined the encapsulation capacity of polymers.

To quantify the interaction strength between a drug and a polymer or biomacromolecules, post‐processing approaches such as Molecular Mechanics/Poisson‐Boltzmann Surface Area (MM/PBSA) and Molecular Mechanics/Generalized Born Surface Area (MM/GBSA) are often employed to estimate binding free energies from classical MD trajectories.^[^ ^275^, ^276^, ^277^ ^]^ One of the key advantages of MM/PBSA and MM/GBSA methods lies in their ability to easily decompose the binding free energy into interpretable components, such as van der Waals, electrostatic, and solvation contributions, thus offering valuable mechanistic insights.^[^ ^275^ ^]^ For example, the co‐assembly mechanism of Evans blue‐conjugated camptothecin (EB‐CPT) with PTX was investigated using AA MD simulations to elucidate their synergistic potential in combination therapy.^[^ ^278^ ^]^ MM/GBSA analyses were employed to evaluate binding affinities, revealing a binding free energy trend of EB‐EB > EB‐CPT > CPT‐CPT in self‐aggregated EB‐CPT clusters in the absence of PTX—highlighting strong EB‐mediated interactions. Notably, the introduction of PTX significantly destabilized EB‐CPT dimers. The dominant forces driving the interaction between EB‐CPT and PTX clusters were found to be van der Waals and hydrophobic interactions. MM/PBSA and MM/GBSA approaches have also been employed to evaluate drug encapsulation efficiency in dendritic nanocarriers. For example, MD and MM/(PB/GB)SA methods were used to assess the stability of generation 4 poly(amidoamine) (PAMAM‐G4) dendrimer complexes carrying four different drugs.^[^ ^279^ ^]^ The results indicated that both neutral and charged PAMAM‐G4 exhibited the most favorable binding free energies for Cromoglicic acid (CRO), Methotrexate (MTX), and Fusidic acid (FUS), driven by a delicate balance between electrostatic and van der Waals interactions. While MTX showed consistent encapsulation efficiency across both neutral and acidic pH conditions, CRO and FUS demonstrated superior binding under acidic conditions compared to neutral or basic environments. However, despite their efficiency and broad applicability, MM/PBSA and MM/GBSA remain highly system‐dependent,^[^ ^280^ ^]^ and exhibit notable limitations, including sensitivity to force field parameters and difficulties in converging entropy contributions.

Currently, enhanced MD techniques are more efficient tools to sample more exhaustively conformational ensembles of AA models, thereby facilitating the accurate calculation of binding free energies.^[^ ^281^ ^]^ For instance, AA MD, MM/(PB/GB)SA, and umbrella sampling approaches have been combined to study the effect of dendrimer chemistry and topology on the solubility and release behavior of phenylbutazone when complexed with PAMAM‐G3 and G4‐diaminobutane‐cored poly(propylene imine) (PPI) dendrimers.^[^ ^282^ ^]^ These methods are particularly useful for investigating drug‐carrier interactions during the release process, which will be discussed in detail in the following section.

### 3.2 Drug Release From Polymer Nanocarriers

The drug release mechanism is a key aspect contributing to the therapeutic efficacy of polymer‐based drug delivery systems. In general, drug release is governed by the physicochemical stability of polymer nanocarriers, and proceeds through processes such as colloidal disassembly, matrix swelling, and/or degradation of the nanoparticle/polymer scaffold. These processes are often triggered by environmental factors, including temperature and pH changes. The interplay between nanoparticle integrity and external stimuli ultimately dictates the spatiotemporal profile of drug release, influencing both bioavailability and therapeutic performance. Current studies have reported the use of computational approaches to investigate drug release kinetics and mechanisms. For example, umbrella sampling with CG models was used to calculate the translocation energy profile of polymer‐drug conjugates such as PEG‐docetaxel (PEG‐Dtxl), oleic acid‐Dtxl (OA‐Dtxl) in a PLGA‐water system to assess drug release kinetics.^[^ ^283^ ^]^ In addition, the DPD method was employed to simulate the effect of drug concentration on drug release kinetics from amphiphilic diblock copolymers containing drug molecules and functional nanoparticles by decreasing the solvent‐drug interaction parameter.^[^ ^284^ ^]^ The results showed that the increase in drug concentration increased the drug release rate. Considering that drug release process can be triggered either by exploiting specific characteristics of disease tissue (i.e., endogenous stimuli), such as over‐expression of certain enzymes in pathological cells, acidic environment of cancer cells, or inflammation‐induced local temperature increase, or by using external physical stimuli (i.e., exogenous stimuli), such as light, ultrasound, or magnetic field,^[^ ^285^, ^286^, ^287^ ^]^ the rational design of stimuli‐responsive polymer‐based drug delivery systems has become very attractive,^[^ ^6^, ^288^ ^]^ and a growing number of computational studies have investigated how various environmental conditions, including pH, salt concentration, temperature, and specific enzymes, can destabilize polymer‐based nanocarriers.^[^ ^289^, ^290^ ^]^

#### 3.2.1 Temperature‐Responsive Polymer Nanoparticles

**Figure 8:**
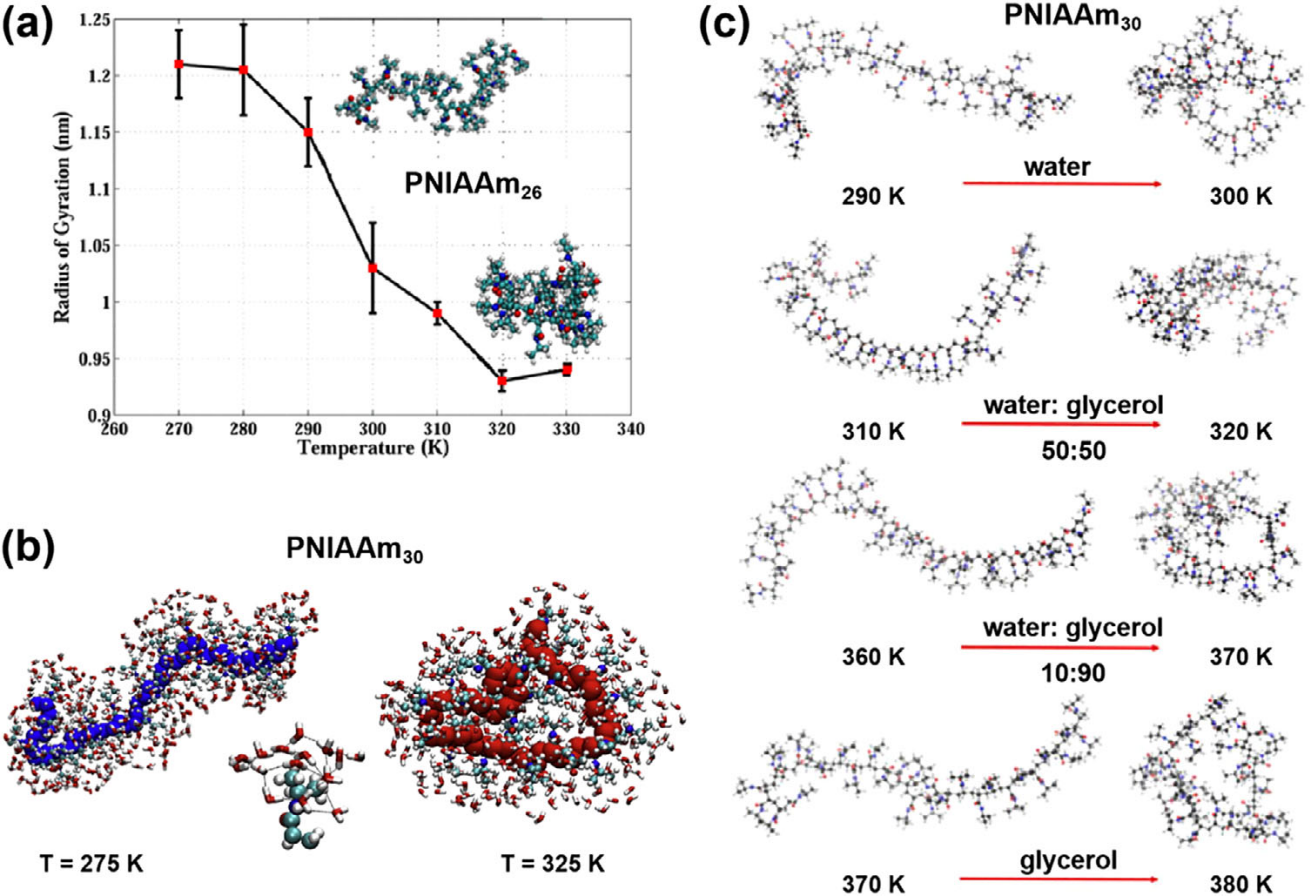
a) Temperature‐dependent radius of gyration (Rg) of a single chain of PNIPAAm. Reproduced with permission.^[^ ^294^ ^]^ Copyright 2017, American Chemical Society. b) First hydration shell of PNIPAAm_30_ at 275 K and 325 K, with the latter showing that water is expelled from the core of the polymer globule. The inset highlights a cage‐like water structure around the isopropyl group of a PNIPAAm chain at 275 K. Reproduced with permission.^[^ ^89^ ^]^ Copyright 2017, Elsevier. c) Instantaneous coil and globule conformation of PNIPAAm_30_ at temperatures below and above their LCST in four solvents: water, water‐glycerol (50:50 v/v), water‐glycerol (10:90 v/v), and glycerol (top to bottom). Reproduced with permission.^[^ ^308^ ^]^ Copyright 2023, Hopkins and Blaisten‐Barojas.

Temperature is a stimulus commonly used to trigger drug release from polymer‐based nanocarriers.^[^ ^4^ ^]^ Thermosensitive polymers exhibit a transition from a hydrated to a dehydrated state, that is, from a homogeneous dissolved state to a heterogeneous two‐phase state, in response to a small change in temperature. Thermosensitive polymers exhibit either a lower critical solution temperature (LCST), that is, soluble below a critical temperature and insoluble above, or an upper critical solution temperature (UCST), which is the opposite behavior. LCST polymers, such as poly(*N*‐isopropyl acrylamide) (PNIPAAm) with a phase transition temperature at ≈32 °C,^[^ ^291^ ^]^ have attracted great interest in the design of thermosensitive polymer‐based drug delivery systems.^[^ ^292^, ^293^ ^]^ Interestingly, given the intrinsic relationship between the volume phase transition of LCST polymer‐based micelles/nanoparticles and the temperature‐dependent compactness of the chains, recent theoretical simulations (primarily conducted at the atomistic scale) have mainly focused on four aspects of the latter. The first aspect is the conformational dynamics of single polymer chains. AA MD simulations with popular force fields (e.g., AMBER or OPLS), coupled with 3‐site water models (e.g., SPCE or TIP3P), have been shown to be an effective method for capturing the coil‐to‐globule conformation change by monitoring the radius of gyration of PNIPAAm single chains as the function of temperature (**Figure** **8**a).^[^ ^294^, ^295^, ^296^ ^]^ The second aspect highlighted the crucial role of hydrogen bond interactions for LCST polymers.^[^ ^297^, ^298^, ^299^, ^300^, ^301^ ^]^ The results of AA simulations have shown that extended polymer conformations were stabilized by hydrogen bonds between water and the polar groups of the polymer at low temperature. Conversely, at high temperature, these hydrogen bonds were weakened and the hydrophobic interactions between the apolar groups of the polymer become dominant, leading to globular conformations (Figure 8b).^[^ ^89^ ^]^ More recently, similar conclusions have been drawn from atomistic MD simulations of other LCST polymers, such as poly(N‐vinylcaprolactam) (PVCL),^[^ ^302^ ^]^ poly(2‐dialkylaminoethyl methacrylate)s,^[^ ^303^ ^]^ *γ*‐substituted poly(*ϵ*‐caprolactone),^[^ ^304^ ^]^ poly(*N*‐n‐propylacrylamide) (PNnPAAm, a structural isomer of PNIPAAm).^[^ ^172^ ^]^ The third aspect concerned the impact of various factors on the LCST property, such as the presence of salts,^[^ ^305^ ^]^ the polymer chain length,^[^ ^306^ ^]^ and solvent effect (Figure 8c).^[^ ^307^, ^308^ ^]^ The simulations carried out matched the experimental results perfectly. The last aspect was focused on the study of thermosensitive polymer‐based drug delivery systems.^[^ ^170^, ^294^ ^]^ AA simulations have shown that drugs were generally encapsulated in a single collapsed copolymer chain above the cloud point, which then extended with decreasing temperature, resulting in an abrupt drug release.

**Figure 9:**
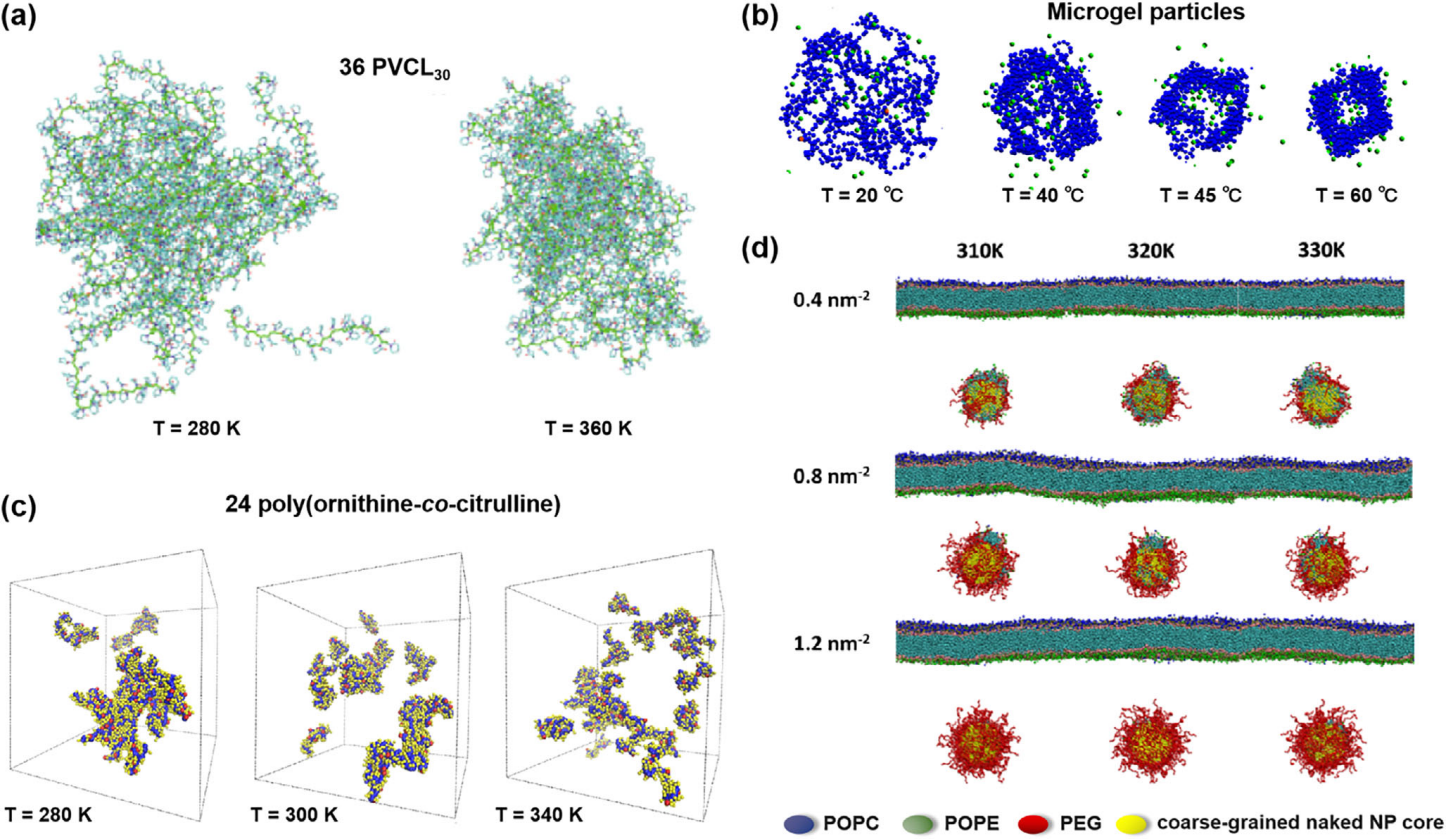
a) Density of an aggregate of 36 chains of PVCL_30_ at 280 and 360 K. Reproduced with permission.^[^ ^302^ ^]^ Copyright 2019, Elsevier. b) Snapshots of the cross‐section of microgel particles from the swollen state to the shrunken state as the temperature increases. The monomers and ions are shown in blue and green, respectively. Reproduced with permission.^[^ ^310^ ^]^ Copyright 2015, American Chemical Society. c) Conformational change of 24 chains of poly(ornithine‐*co*‐citrulline) at three different temperatures. Reproduced with permission.^[^ ^90^ ^]^ Copyright 2021, American Chemical Society. d) Effect of temperature and PEG density on the permeability of nanoparticles across a biomembrane. Reproduced with permission.^[^ ^313^ ^]^ Copyright 2017, Elsevier.

However, due to the high computational cost of AA simulations, far fewer studies have been reported about the collective behavior of multiple LCST polymer chains using this approach, thus limiting the understanding of the polymer aggregation processes and the mechanism of drug release from these nanocarriers. Representative simulation studies of LCST polymers were performed on 3‐5 chains of PNIPAAm,^[^ ^294^ ^]^ 4 chains of PNnPAAm,^[^ ^172^ ^]^ 18 chains of PNIPAAm,^[^ ^309^ ^]^ as well as on 36 chains of PVCL.^[^ ^302^ ^]^ Overall, these studies highlighted the fact that, when the temperature was increased, the number of polymer‐water hydrogen bonds decreased in favor of the polymer‐polymer interactions. Nevertheless, it was unclear whether these inter‐chain interactions induced less pronounced polymer compactness in aggregates than in single‐chain simulations, or conversely, whether the aggregates could be considered as globules of collapsed chains.^[^ ^172^, ^309^ ^]^ It is interesting to note that simulations of 18 PNIPAAm chains^[^ ^309^ ^]^ and 36 PVCL_30_ chains^[^ ^302^ ^]^ clearly showed a significant increase in the density of polymer aggregates with increasing temperature (**Figure** **9**a), in line with the known volume phase transition of LCST polymers. Also worth mentioning is a MC study of 206 PNIPAAm chains based on a CG model, in which hydrophobic beads interacted with each other via a phenomenological potential whose depth increased with temperature.^[^ ^310^ ^]^ The simulations were able to reproduce the shrinking of polymer aggregates with increasing temperature and showed that the swollen microgel at low temperature had a rather homogeneous structure, whereas inner holes were formed in the shrunk state at high temperature (Figure 9b). In a recent study, significant advancement was achieved in modeling the LCST behavior of polypeptides using CG simulations.^[^ ^311^ ^]^ Leveraging the Martini 3 force field by introducing temperature‐dependent modifications to the Lennard‐Jones (LJ) potentials between solute apolar or charged beads and water beads, they successfully reproduced the LCST behavior of multi‐chain systems. This tailored adjustment enabled CG model to capture the polypeptide phase behavior as a function of temperature, thereby demonstrating the applicability of the Martini 3 framework for simulating LCST phenomena of peptide‐based systems. Although these reports currently only emphasized the importance of considering the collective behavior of several polymer chains to better understand the temperature‐induced morphology change of polymer aggregates, they nonetheless provide a valuable theoretical foundation for the rational design of temperature‐responsive drug delivery systems.

Compared with LCST polymer nanocarriers, for which the collapse of polymer chains above the cloud point often leads to partial drug entrapment, UCST polymer nanocarriers are expected to completely release their drug payload above the transition temperature by solubilization of the polymer chains. Yet, in contrast to LCST polymers, UCST polymer‐based nanocarriers have received much less attention, probably due to the much smaller number of UCST polymers with relevant transition temperatures for biomedical applications (e.g., from physiological conditions up to ≈43 °C).^[^ ^4^, ^312^ ^]^ Consequently, very few computational studies on the conformational dynamics of UCST polymer‐based nanocarriers have been reported so far.

Interestingly, two of the pioneered simulation studies on UCST polymers were performed on a single polymer chain in a mixture of organic solvent and water. Using AA MD simulations, a poly(styrylphenyl(tri‐iso‐propylphenyl)borinic acid) (PBA) polymer chain in 7% H_2_O/DMSO solvent mixture showed an increase in the SASA value when the temperature was increased.^[^ ^314^ ^]^ They attributed the compactness of the polymer chain at low temperature to the stability of water molecules in the vicinity of boric acid's hydroxyl groups, which can form hydrogen‐bonding bridges between the polymer's pendant chains. At high temperature, water molecules were expelled, and the polymer chain was solvated by DMSO molecules only, inducing a more extended conformation. Similarly, the temperature‐dependent conformational behavior of a single chain of polyacrylamide (PAAm) in 50% alcohol (methanol or ethanol)‐water solutions was studied with AA MD simulations.^[^ ^315^ ^]^ The results indicated an increase in the radius of gyration of the polymer with increasing temperature, in agreement with the experimentally observed UCST behavior. Similarly, the polymer compactness at low temperature was attributed to the formation of stable water‐bridges between the pendent acrylamide units, which are broken at high temperature, favoring extended conformations solvated by alcohol molecules.

The important role of polar/electrostatic interactions in UCST polymers was also emphasized in AA simulations of 4 chains of cationic poly(methacrylate‐*g*‐pentaarginine) P(MA‐R5) in water.^[^ ^316^ ^]^ The simulated polymer chains were observed to aggregate into dimers and trimers at low temperature (20 °C), which were stabilized by inter‐chain stacking interactions between the arginine guanidinium (Gdm+) groups. It should be noted that these counterintuitive cation‐cation interactions in water have been demonstrated theoretically and experimentally in previous studies,^[^ ^317^, ^318^ ^]^ and have been shown to be responsible for the self‐aggregation of deca‐arginine (R10) peptides.^[^ ^319^ ^]^ The P(MA‐R5) simulations showed that the number and lifetime of these Gdm+/Gdm+ stacking at high temperature (85 °C) were approximately half that at low temperature, suggesting a dissolution of the aggregates. Similar AA MD studies were carried out on 5 chains of poly(acrylamide‐*co*‐vinyl acetate) copolymers in water.^[^ ^173^ ^]^ The results showed a decrease in the number of inter‐chain hydrogen bonds concomitantly with an increase in polymer‐water bonds as the temperature increases.

Although the aforementioned AA simulations have facilitated a more comprehensive understanding of the physicochemical forces that govern the UCST polymer properties, they are not yet sufficiently reliable to be used for predicting the transition temperatures and guiding the optimization of UCST polymers with potential for biomedical applications. To this end, it is probably more efficient to run CG simulations of several polymer chains, allowing observation of their aggregation and dissolution processes as a function of temperature. A representative example of this approach was illustrated by the study of the UCST behavior of poly(ornithine‐*co*‐citrulline) copolymers.^[^ ^90^ ^]^ CG simulations of 24 chains composed of 8 ornithine and 72 citrulline residues at 5 different temperatures were performed to reproduce the copolymer aggregation‐dissolution dynamics at low and high temperatures (Figure 9c). Interestingly, the theoretical estimation of the phase transition temperature was found to be in close agreement with the experimental results.^[^ ^90^ ^]^ Despite the lack of computational studies on drug‐loaded nanocarriers based on UCST polymer, the progression of computational techniques for these temperature‐sensitive macromolecules will undoubtedly facilitate future developments in controlled release drug delivery systems.

In this regard, a few CG MD simulations were used to model the drug release and delivery processes of polymer nanoparticles. For instance, umbrella sampling was applied to study Ptx release efficiency from PEG‐PLA polymer micelles at different temperatures.^[^ ^188^ ^]^ The PMF profiles revealed that elevated temperatures and higher ethanol concentrations significantly reduced the energy barriers for Ptx release, indicating that these conditions destabilize the micellar structure and promote enhanced drug release. In a complementary study, nanoparticles with varying PEG densities were simulated during translocation across an asymmetric lipid bilayer at three different temperatures to evaluate the impact of temperature and PEG grafting density on their membrane‐crossing capability. The change in the PMF during translocation was smaller at 330 K than at 310 K, demonstrating that a higher temperature could lower the free energy barrier of their translocation process (Figure 9d).^[^ ^313^ ^]^

#### 3.2.2 pH‐Responsive Polymer Nanoparticles

pH‐sensitive polymer nanocarriers have also been identified as potent stimuli‐responsive drug delivery systems, with the potential to achieve targeted and controlled drug release, due to the specific pH of pathological tissues, for instance, during inflammation, infection, or cancer. One strategy for designing pH‐responsive polymer nanoparticles is to select a polymer with pH‐sensitive chemical groups, such as acetal, hydrazone, vinyl ester,^[^ ^320^, ^321^ ^]^ etc. Representative examples of pH‐responsive polymers include poly(acrylic acid) (PAA),^[^ ^322^ ^]^ as well as dendritic polymers such as PAMAM^[^ ^252^, ^323^ ^]^ and PPI,^[^ ^324^ ^]^ which are widely employed in the design of drug delivery systems. An alternative approach is to encapsulate pH‐sensitive drugs with weak acid or base groups, such as famotidine (pKa = 7.1), indomethacin (pKa = 5.7), or amphotericin B (pKa = 5.7 and 10.0).^[^ ^325^ ^]^ The idea common to both approaches is that a subtle variation in pH will change the protonation states and charges of pH‐sensitive chemical groups, which in turn will induce a destabilization of the nanoparticle supramolecular organization. As with temperature‐sensitive nanocarriers, numerous simulation studies have been carried out to better understand the pH‐induced drug release mechanism. In this review, these investigations were broadly categorized into the following three areas.

**Figure 10:**
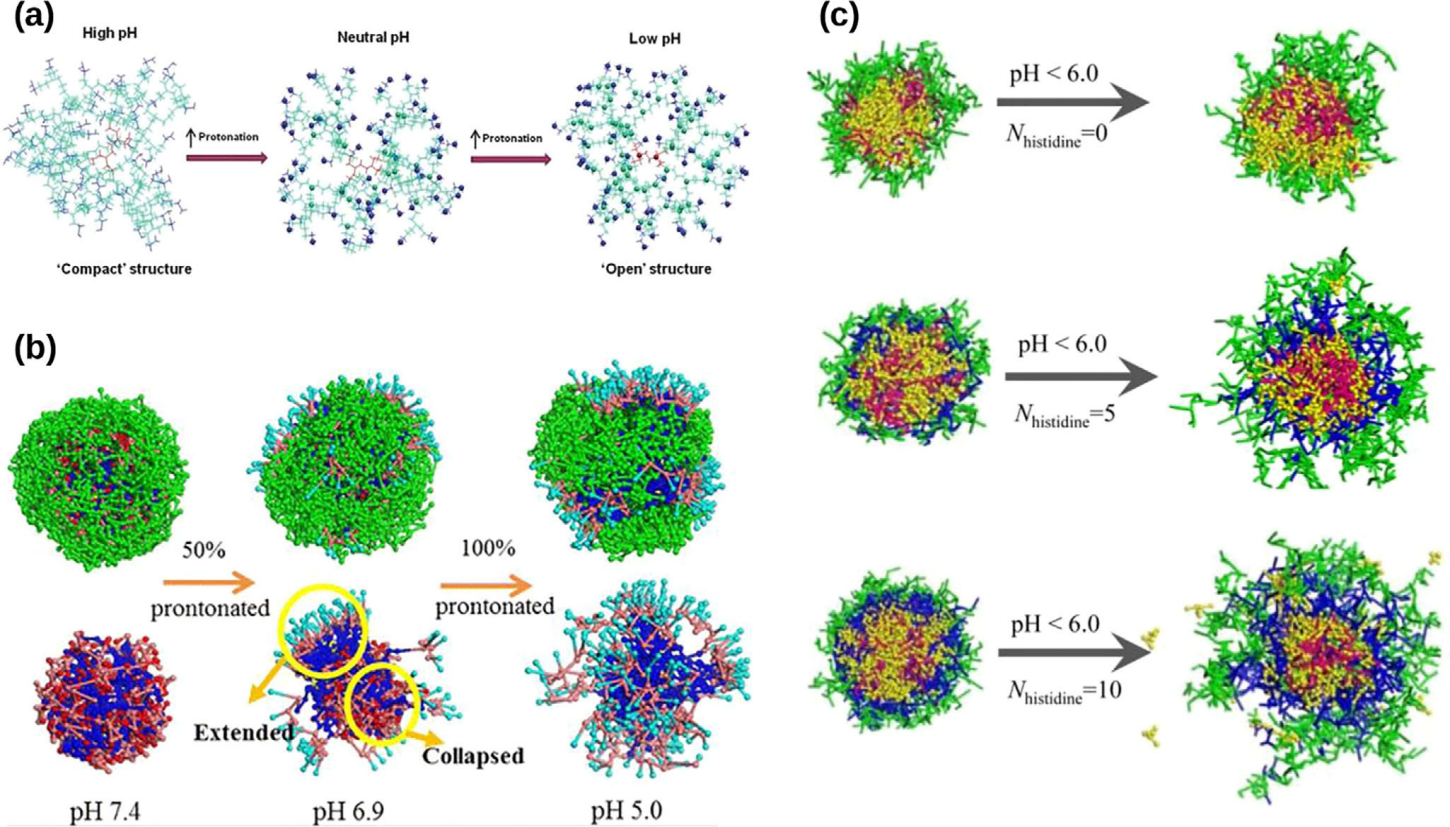
a) pH‐dependent density of PPI dendrimer nanoparticle in AA model. Reproduced with permission.^[^ ^282^ ^]^ Copyright 2013, the Royal Society of Chemistry. b) The conformational dynamics of pH‐sensitive 4AS‐PCL‐*b*‐PDEAEMA‐*b*‐PPEGMA micelles at different pH in DPD model (blue: PCL, red: PDEAEMA (non‐protonated), cyan: PDEAEMA‐H^+^, green: PPEGMA). Reproduced with permission.^[^ ^149^ ^]^ Copyright 2013, American Chemical Society. c) The morphology of DHA‐His*_x_*‐Lys_10_ (*x* = 0, 5, 10) block copolymer nanoparticle with pH in DPD model. Reproduced with permission.^[^ ^167^ ^]^ Copyright 2015, American Chemical Society.

- pH‐sensitive polymer‐based drug delivery system, as a major design strategy: Among these theoretical investigations, the conformations and interactions of ethylenediamine cored PPI dendrimers (PPI*^EDA^*) with famotidine or indomethacin drugs were investigated at different pH.^[^ ^282^ ^]^ The AA MD simulations demonstrated that the amine groups of the polymer, which are non‐protonated and non‐charged at high pH, allowed a rather dense structure of the dendrimer nanoparticle, while at low pH, they were protonated and positively charged, and the electrostatic repulsion induced a more open structure with several cavities in which water molecules were able to penetrate (**Figure** **10**a). Additionally, by using umbrella sampling simulations, the authors computed the FEP (or PMF) of dendrimer‐drug complexes as a function of their distance. The results showed that, unlike at high or neutral pH, the dendrimer's open structure at low pH induced spontaneous release (no energy barrier) of encapsulated drugs. AA and CG MD simulations have also been employed to investigate the pH‐responsive release mechanism of Ptx from chitosan‐Eudragit‐based nanocarriers.^[^ ^326^ ^]^ These simulations revealed that the nanocarriers maintain structural integrity under alkaline conditions, whereas exposure to acidic environments disrupts hydrogen bonding and compromises structural stability, ultimately triggering nanocarrier deformation and Ptx release. Mesoscale DPD simulations were also performed to investigate the pH‐sensitive morphology of Dox‐loaded poly(*ε*‐caprolactone)‐*b*‐poly(2‐(diethylamino) ethyl methacrylate)‐*b*‐poly(poly(ethylene glycol) methyl ether methacrylate) (4AS‐PCL‐*b*‐PDEAEMA‐*b*‐PPEGMA) micelles at larger spatial and temporal scales.^[^ ^149^ ^]^ The simulations showed that the micelles had a well‐defined core‐mesosphere‐shell three‐layer structure at physiological pH (Figure 10b). However, at lower pH, the pH‐sensitive PDEAEMA blocks became protonated and tend to move toward the nanoparticle surface, inducing micelle swelling and the formation of surface cracks, which could promote the release of the encapsulated drugs. In a similar DPD study, the morphology of Dox‐loaded micelles composed of block polymers made of docosahexanoic acid (DHA) and His_x_‐Lys_10_ peptides (*x* = 0, 5, 10) was investigated.^[^ ^167^ ^]^ At pH > 6, the DHA‐His*_x_*‐Lys_10_ polymers self‐assembled into dense core‐shell micelles in which the DHA, His, and Lys were located in the core, the middle, and the external layer, respectively (Figure 10c). Like the previous system, a decrease in pH led to protonation of His residues, inducing micelle swelling, which can potentially facilitate drug release.
- pH‐responsive bonds in polymer drug delivery system: MD simulations have been employed to investigate the adsorption behavior and dynamic interactions of poly(β‐malic acid)‐doxorubicin (PMLA‐Dox) conjugates linked via either an amide bond (PMLA‐ami‐Dox) or a pH‐sensitive hydrazone bond (PMLA‐hz‐Dox), as well as free Dox, on GO surfaces under neutral and acidic conditions.^[^ ^327^ ^]^ The simulations revealed that free Dox molecules readily adsorbed onto the GO surface, primarily driven by π‐π stacking and hydrogen bonding interactions. Interestingly, Dox conjugated via hydrazone linkages (PMLA‐hz‐Dox) exhibited stronger interactions with GO compared to their amide‐linked counterparts, resulting in more stable drug‐loaded nanocarriers. Under acidic conditions, the binding affinity between PMLA‐hz‐Dox conjugates and the GO surface was reduced relative to neutral pH, facilitating partial desorption and pH‐triggered drug release.
- Membrane penetration of pH‐sensitive drug delivery system: The cellular uptake behavior of MTX‐loaded nanoparticles grafted with hydrophilic γ‐polyglutamic acid (MTX‐SS‐γ‐PGA), a drug‐carrier system exhibiting pH‐dependent solubility, has been investigated using AA MD combined with umbrella sampling.^[^ ^328^ ^]^ The results demonstrated that MTX‐SS‐γ‐PGA nanoparticles exhibit stronger interactions with the head groups of lipid bilayers under tumor‐mimicking acidic conditions (pH = 6.4) compared to physiological pH, attributed to enhanced charge fluctuations and increased electrostatic interactions. PMF profiles further revealed that the energy barrier for membrane penetration is significantly reduced at lower pH, indicating that acidic environments facilitate more efficient translocation of the drug‐loaded nanoparticles across the membrane. In addition, MD simulations have also been utilized to study the membrane penetration behavior of GO decorated with a pH‐sensitive polymer prodrug, in which Dox was conjugated to polyethyleneimine (PEI) via a pH‐sensitive cis‐aconitic anhydride (CA) linker.^[^ ^329^ ^]^ The simulation results revealed that Dox was effectively released under acidic conditions. Moreover, GO demonstrated strong potential as a nanocarrier capable of facilitating the translocation of polymer prodrugs across lipid membranes. Notably, the interactions between GO and the prodrug were sufficiently robust to prevent premature drug leakage during transport, underscoring the viability of GO‐based systems for stable and stimuli‐responsive drug delivery.

In addition to MD simulations, alternative computational techniques have been employed to investigate the behavior of pH‐responsive polymer‐based nanocarriers. For instance, MC methods were used to examine a Ptx/β‐cyclodextrin (β‐CD) nanocomplex composed of polyvinyl alcohol (PVA), with a focus on the host‐guest interactions between Ptx and β‐CD.^[^ ^330^ ^]^ The simulations revealed favorable binding affinities and pH‐dependent Ptx release characteristics. Notably, under acidic conditions, the Ptx/β‐CD nanocomplex demonstrated enhanced drug release efficiency, alongside strong binding interactions, highlighting its potential as a stimuli‐responsive delivery platform for controlled drug release in the tumor microenvironment.

#### 3.2.3 Enzyme‐Sensitive Polymer Prodrugs

Taking advantage of endogenous enzymes to trigger controlled drug release from polymer‐based drug delivery systems is another important and commonly used strategy for achieving targeted therapy of diseased cells or tissues. Indeed, specific enzymes generally involved in cell growth, motility, angiogenesis, or endocytosis are often found in abnormally high concentrations in various pathological cells, particularly in cancer cells. This feature can be exploited to specifically deliver drugs to these cells while avoiding delivery to healthy ones. Thanks to the high specificity of enzymes, this aim can be achieved by designing polymer prodrugs^[^ ^6^, ^49^ ^]^ with specific chemical linkers that can be recognized and cleaved by overexpressed enzymes in targeted tissues. Specific enzymes that have been used to design enzyme‐sensitive polymer prodrug delivery systems include urease, lipase, histone deacetylase, oxidase, and peroxidase.^[^ ^331^, ^332^, ^333^, ^334^, ^335^ ^]^

Molecular recognition and cleavage efficacy of the polymer‐drug linker by specific enzymes depends on various parameters such as the nature and physicochemical properties of the linker, the drug, and the polymer. Consequently, the use of molecular simulations is also very useful in the early stages of drug delivery system development, as it can help to better understand the mechanism of enzymatic‐mediated drug release and to predict the drug release and their efficacy. In this context, the supramolecular organization of self‐assembled Gem‐PI and Ptx‐PI polymer prodrug nanoparticles was studied by CG MD simulations, and it was shown that the localization of the linkers was correlated to their hydrophilicity.^[^ ^268^ ^]^ Based on the solvent accessible surface area (SASA) of the various cleavage sites of the linkers, this model was further used to predict the drug release efficiency and to develop a new linker capable of enhancing the cytotoxicity of the nanoparticles.^[^ ^189^ ^]^

In a different approach, the impact of the drug and polymer natures upon the cleavage of the linker for various polymer prodrugs by human carboxylesterase 2 (hCE2) was investigated. The polymer prodrugs were constructed from four different drugs (Gem, 7‐ethyl‐10‐hydroxycamptothecin (SN38), Dox, and Ptx) covalently linked through an ester moiety to three different polymers (PCL, PGA, and PLA).^[^ ^336^ ^]^ By comparing ester cleavage free energy profiles computed by quantum mechanics/molecular mechanics (QM/MM) simulations, the authors highlighted that drug size was decisive for ester cleavage kinetics. The results showed that small drugs (Gem) were more efficiently released from the prodrug than bigger ones (Ptx). Although costly in terms of computational time, these simulations have led to a better understanding of the factors influencing the drug release efficiency from polymer prodrugs, thereby offering insights into the rational design of polymer prodrugs for enzyme‐sensitive drug delivery systems.

## 4 ML and MD: A look at the Emerging Opportunities and Challenges

The convergence of artificial intelligence (AI) and MD is rapidly expanding the landscape of molecular simulations. AI, particularly through ML and deep learning techniques, offers promising strategies to overcome longstanding challenges in MD, including the trade‐off between accuracy and computational cost, and the limited access to long‐timescale phenomena. Among the most impactful developments is the emergence of ML‐derived interatomic potentials that approach quantum mechanical accuracy while significantly reducing computational cost.^[^ ^337^ ^]^ In the realm of polymer and drug delivery research, ML and deep learning models have demonstrated the ability to predict critical material and formulation properties, including the composition‐microstructure relationship of polymer nanocomposites, glass transition temperatures (*T_g_*) of polymers, as well as the design and therapeutic strategies of inorganic NPs in relation to their preclinical evaluation.^[^ ^338^, ^339^, ^340^ ^]^ For instance, the inverse design of polymers with extreme properties (e.g., *T_g_* > 500 K, and bandgap *E_g_* > 6 eV) has been facilitated by genetic algorithms coupled with ML predictors.^[^ ^339^ ^]^ Similarly, in the context of nanocarriers, ML models have been employed to decode how drug properties (e.g., molecular weight, solubility, drug‐to‐lipid ratio) and preparation parameters (e.g., preparation temperature and method) influence key liposome performance metrics, such as particle size, polydispersity index (PDI), zeta potential, and encapsulation efficiency, to guide efficient liposome formulation design.^[^ ^341^ ^]^

Despite these advances, the deployment of data‐driven models faces inherent challenges—most notably, the scarcity of high‐quality training datasets and the “black‐box” nature of model decision‐making, which limits interpretability.^[^ ^85^ ^]^ In contrast, MD simulations offer physics‐based “clear‐box” models rooted in well‐established physical laws, thereby providing mechanistic insights into system behavior. Thus, the integration of MD with ML methodologies has been a good strategy to combine the interpretability and theoretical rigor of MD with the pattern recognition and predictive efficiency of ML.^[^ ^85^ ^]^ A representative example of this synergy is provided by Houshmand et al., who integrated MD and ML techniques to predict the SASA value of nanoparticles, a critical parameter in nanocarrier design.^[^ ^342^ ^]^ Their approach yielded a 25% improvement in predictive accuracy and a remarkable 300‐fold enhancement in computational efficiency over conventional methods. This integrated framework not only outperforms existing SASA prediction techniques but also exhibits strong scalability and robustness, making it suitable for broader applications in drug discovery and materials science. Looking forward, the fusion of MD with ML is poised to become a paradigm‐shifting approach in computational nanomaterials research—enabling rational design, accelerating discovery, and ultimately transforming the development of next‐generation polymer‐based drug delivery systems.

## 5 Conclusion and Outlook

In this review, we have presented several common computational techniques to study polymer‐based drug delivery systems, including AA MD, CG MD, DPD, MC, as well as enhanced MD simulation techniques. We then reported some computational investigations of the self‐assembly and supramolecular organization of polymer nanocarriers for drug delivery applications, including drug‐loaded polymer micelles and polymer prodrug nanoparticles. We further discussed how modeling approaches can rationalize the drug loading and release from polymer drug delivery systems. In particular, we described some simulations studies which aim at better understanding how physical, chemical, or biological stimuli (e.g., temperature, pH, and enzymes) can trigger the drug release. By highlighting representative applications and mechanistic insights, this review aims to provide guidance and reference for experimentalists and early‐stage interdisciplinary researchers interested in incorporating simulation technologies into the design and analysis of advanced drug delivery systems.

Despite the visible advances in computational techniques within this field, several critical challenges and opportunities remain. A key limitation is the trade‐off between chemical resolution and spatiotemporal scale, while AA simulations offer molecular‐level detail, they are computationally prohibitive for accessing biologically relevant timescales and system sizes. Conversely, CG and mesoscale models allow simulation of large systems over longer times but often sacrifice chemical specificity. The integration of multiscale modeling frameworks that can bridge different levels of resolution without losing essential physical accuracy, but demands careful calibration and high compatibility across model parameters to preserve physical accuracy and continuity. Looking forward, the integration of ML and MD with conventional computational chemistry offers a promising new paradigm. These data‐driven methods can be employed to predict physicochemical properties, explore vast chemical design spaces, and optimize nanocarrier architectures in a high‐throughput manner. When trained on reliable simulation and experimental datasets, ML models can accelerate the inverse design of polymer‐drug systems with targeted properties, thereby reducing the reliance on trial‐and‐error experimentation.

In conclusion, computational simulations are not only analytical tools but also predictive engines that appear to be invaluable tools for providing new ideas for designing next‐generation polymer‐drug nanocarriers. By combining MD with ML approaches and experimental feedback, the field is well‐positioned to overcome current limitations and contribute significantly to the development of personalized and precision drug delivery strategies.

## Conflict of Interest

The authors declare no conflict of interest.
